# Optimized design of single-cell RNA sequencing experiments for cell-type-specific eQTL analysis

**DOI:** 10.1038/s41467-020-19365-w

**Published:** 2020-10-30

**Authors:** Igor Mandric, Tommer Schwarz, Arunabha Majumdar, Kangcheng Hou, Leah Briscoe, Richard Perez, Meena Subramaniam, Christoph Hafemeister, Rahul Satija, Chun Jimmie Ye, Bogdan Pasaniuc, Eran Halperin

**Affiliations:** 1 Department of Computer Science, University of California Los Angeles, 404 Westwood Plaza, Los Angeles, CA 90095 USA; 2 Bioinformatics Interdepartmental Program, University of California Los Angeles, 611 Charles E. Young Drive East, Los Angeles, CA 90095 USA; 3 Department of Pathology and Laboratory Medicine, David Geffen School of Medicine, University of California Los Angeles, 10833 Le Conte Ave, Los Angeles, CA 90095 USA; 4 Institute for Human Genetics, University of California San Francisco, 513 Parnassus Avenue, San Francisco, CA 94143 USA; 5 Bakar Computational Health Sciences Institute, University of California San Francisco, 550 16th Street, San Francisco, CA 94158 USA; 6 Division of Rheumatology, Department of Medicine, University of California San Francisco, 513 Parnassus Avenue, San Francisco, CA 94143 USA; 7 Bioinformatics Program, University of California San Francisco, 513 Parnassus Ave, San Francisco, CA 94143 USA; 8 New York Genome Center, 101 Avenue of the Americas, New York, NY 10013 USA; 9 Center for Genomics and Systems Biology, New York University, 12 Waverly Place, New York, NY 10003 USA; 10 Department of Human Genetics, David Geffen School of Medicine, University of California Los Angeles, 6506 Gonda Center, Los Angeles, CA 90095 USA; 11 Department of Computational Medicine, David Geffen School of Medicine, University of California Los Angeles, Room 5303 Life Sciences, Los Angeles, CA 90095 USA; 12 Department of Anesthesiology and Perioperative Medicine, David Geffen School of Medicine, University of California Los Angeles, 757 Westwood Plaza, Los Angeles, CA 90095 USA; 13 Institute of Precision Health, University of California, Los Angeles, CA USA

**Keywords:** Computational models, Statistical methods, Quantitative trait

## Abstract

Single-cell RNA-sequencing (scRNA-Seq) is a compelling approach to directly and simultaneously measure cellular composition and state, which can otherwise only be estimated by applying deconvolution methods to bulk RNA-Seq estimates. However, it has not yet become a widely used tool in population-scale analyses, due to its prohibitively high cost. Here we show that given the same budget, the statistical power of cell-type-specific expression quantitative trait loci (eQTL) mapping can be increased through low-coverage per-cell sequencing of more samples rather than high-coverage sequencing of fewer samples. We use simulations starting from one of the largest available real single-cell RNA-Seq data from 120 individuals to also show that multiple experimental designs with different numbers of samples, cells per sample and reads per cell could have similar statistical power, and choosing an appropriate design can yield large cost savings especially when multiplexed workflows are considered. Finally, we provide a practical approach on selecting cost-effective designs for maximizing cell-type-specific eQTL power which is available in the form of a web tool.

## Abstract (web summary)

Single cell RNA-sequencing can be a powerful approach to characterizing cell composition in a population of cells but is thought to be too expensive for population-scale analyses. Here, the authors show how lower coverage of more samples can increase the power to detect cell-type-specific eQTL.

## Introduction

Massively parallel single-cell RNA sequencing (scRNA-Seq) has been increasingly used over the past few years as a powerful alternative to bulk RNA-Seq. Key advantages of scRNA-Seq over bulk methods are the ability to reveal complex and rare cell populations^1–5^, uncover regulatory relationships between genes^6–8^, and track the trajectories of distinct cell lineages in development^9,10^. While the first scRNA-Seq dataset in 2009 consisted of only eight cells^11^, the number of cells in a typical experiment today is approaching tens or even hundreds of thousands^12,13^.

Expression quantitative trait locus (eQTL) mapping is a widely used tool in functional genomics used to identify mechanisms underlying the genotype-to-disease connection^14,15^ and genetic regulation of gene expression^16,17^. Traditionally, gene expression measurements used in eQTL studies are obtained from bulk measurements such as expression arrays or RNA-Seq^14,18^. However, cell-type specificity of eQTLs^19^ suggests that bulk approaches are suboptimal if the tissue of interest is composed of multiple cell types. The ability to simultaneously estimate cellular composition and state using scRNA-Seq creates an enormous opportunity to apply scRNA-Seq to large population cohorts to detect subtle shifts in single-cell transcriptomics associated with population level variation (e.g., genetics and/or disease status). One of the main limitations of scRNA-Seq had been its high cost, which with the development of cost-effective multiplexed workflows, has been significantly mitigated enabling the broader adoption of population-scale scRNA-Seq and cell-type-specific eQTL studies (ct-eQTL)^20–22^.

Ct-eQTL mapping critically depends on assaying many individuals, which is needed in order to achieve sufficient statistical power for detecting true associations. Therefore, despite the recent considerable drop in sequencing cost^23^, the total expense of a large-sample single-cell study can still be prohibitively high^24^. ScRNA-Seq measures transcript abundances for each cell. Obtaining highly accurate single-cell expression profiles is important for downstream analyses. For example, accurate single-cell expression profiles are required to quantify variance within a homogeneous population of cells. Such analyses usually require a high-coverage sequencing (0.5–3 million reads per cell)^25,26^. On the other hand, quantitative genetic analyses such as ct-eQTL mapping, do not necessarily require precise single-cell gene expression estimates. Instead, the average gene expression estimates within a cell type are used in these settings. In the case of noisy single-cell estimates, it is still possible to obtain an adequate level of accuracy given a large enough number of cells. In other words, cell-type-specific gene expression can be quantified accurately by high-coverage RNA-Seq of a single cell or by shallow coverage of multiple cells of a given cell-type followed by aggregation of the information within a cell type. Thus, low-coverage sequencing is a promising approach to infer cell-type-specific gene expression profiles.

The impact of per-cell read coverage on downstream analyses such as cell-type identification^10,27^ and dimensionality reduction^28^ has been studied from both practical and theoretical perspectives. A recent study^29^ investigated the trade-off between read coverage and the number of cells under a fixed budget constraint optimizing for recovering the true underlying gene expression distribution. The main result in ^29^ suggests that only one read per gene per cell is sufficient to accurately recover gene expression distributions, but it does not provide any practical guidelines on how to choose the number of reads per cell nor the number of cells per sample to maximize the power for detecting ct-eQTLs. In addition, that study does not consider critical factors such as the number of sequenced individuals, the impact of cell-type identification, and sample multiplexing to reduce library preparation cost. Sample multiplexing refers to pooling cells from multiple samples for single-cell library preparation at increased throughput. It is possible to demultiplex the pooled samples computationally leveraging sample specific barcodes^30,31^. For example, one of the most widely used methods demuxlet leverages genetic variation captured from the transcriptome of each cell to accurately assign sample identity to each cell^32^.

In this work, we first demonstrate that cell-type-specific gene expression can be accurately inferred with low-coverage single-cell RNA sequencing given enough cells and individuals. Namely, we show that by aggregating reads across cells within a cell type, it is possible to achieve a high average Pearson *R*^2^ between the low-coverage estimates and the ground truth values of gene expression. Second, through extensive simulations starting from real single-cell RNA-Seq data (*N* = 120), we show that by increasing sample size and the number of cells per individual while decreasing coverage, it is possible to reduce the cost of the experiment by half (or even more) while maintaining the same statistical power. Third, we provide a practical guideline for designing ct-eQTL studies which maximizes statistical power. Our results provide a pathway for the design of efficient cell-type-specific association studies that are scalable to large populations.

## Results

### Accurate cell-type-specific gene expression at low-coverage RNA sequencing

**Fig. 1:**
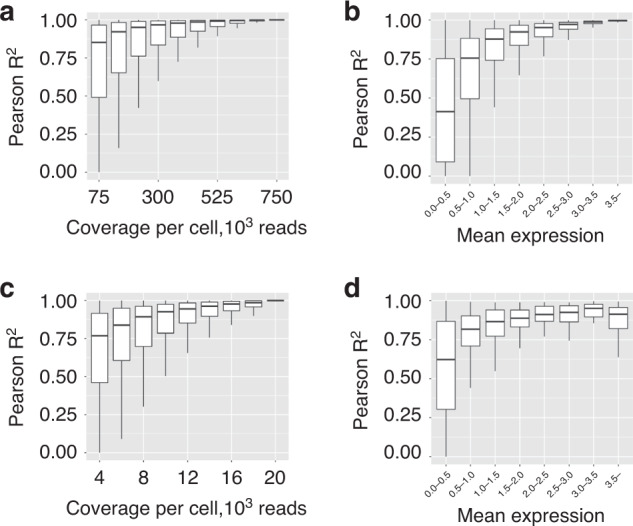
Average *R*^2^ between low-coverage and high-coverage gene expression estimates (Smart-Seq2 dataset, alpha cells). **a** Distribution of Pearson *R*^2^ computed across all the genes at different levels of read coverage, Smart-Seq2 dataset. **b** Distribution of Pearson *R*^2^ at 75,000 reads per cell stratified by the expression level, Smart-Seq2 dataset. **c** Distribution of Pearson *R*^2^ computed across all the genes at different levels of read coverage, 10× dataset. **d** Distribution of Pearson *R*^2^ at 4000 reads per cell stratified by the expression level, 10× dataset. The center line, bounds of box, and whiskers represent mean, 25th to 75th percentile range, and minimum to maximum range in all boxplots.

To accurately quantify gene expression per cell, it is necessary to sequence each cell at a high coverage. However, in ct-eQTL studies, accurate cell-type-specific expression estimates can be achieved with low-coverage sequencing by pooling cells of the same type. To demonstrate this, we used a Smart-Seq2 dataset^33^ consisting of 2209 pancreatic cells obtained from 10 individuals. In this dataset, each cell was sequenced at high coverage (750,000 reads per cell on average), resulting in a reliable estimate of cell-type-specific gene expression. Similar to existing works^10,29,34^, we downsampled the reads uniformly without replacement from the initial dataset. At various levels of coverage, for each cell type, we estimated the Pearson’s *R*^2^ for every gene between the downsampled and the full, gold standard, dataset. Due to the biases of single-cell RNA-Seq technologies, the full, high-coverage dataset is not the ground truth, but it can provide the best attempt at an accurate cell-type-specific measure of gene expression. Therefore, throughout the paper, we will refer to it as the high-coverage dataset. We observe that we can capture most of the expression signal using much lower coverage. For example, 10% of the data (≈75,000 reads per cell) was sufficient to attain ≈70% average *R*^2^ across 24,181 genes in alpha cells (Fig. 1a; see some example genes in Supplementary Fig. 1). This suggests that under idealistic settings of no library preparation cost, the statistical power can be increased by up to tenfold by distributing coverage across many individuals. This is due to the fact that statistical power in an association study is a function of sample size and both the phenotype and genotype measurement accuracy. The power of a study with sample size *N* and estimated phenotype ${\tilde{\mathbf{y}}}$ is approximately the same as the power of a study with sample size *αN* and true phenotypes **y**, where *α* is Pearson *R*^2^ between ${\tilde{\mathbf{y}}}$ and **y**^35,36^. Indeed, let **y** be the high-coverage gene expression vector for a given gene *G* across *N* individuals (i.e., gene expression obtained at high read coverage) and ${\tilde{\mathbf{y}}}$ be the vector of gene expression estimates obtained at low read coverage of the same gene *G* across the same *N* individuals. Let *r* be the Pearson correlation coefficient between **y** and ${\tilde{\mathbf{y}}}$. Let **g** be the genotype at the single-nucleotide polymorphism (SNP) under testing. For simplicity, all the vectors are standardized with zero mean and unit variance. Let *β* and $\tilde \beta$ be the effect sizes of the SNP in the regression on **y** and ${\tilde{\mathbf{y}}}$ correspondingly. Regressing **y** on ${\tilde{\mathbf{y}}}$ we obtain

1 $$\widetilde {\mathbf{y}} = r{\mathbf{y}} + {\mathbf{\epsilon }}.$$

Next, observe that the OLS estimate of $\tilde \beta$, $\widehat {\tilde \beta } = r\hat \beta$. Indeed, let ${\mathbf{\epsilon }}$ be noise random variables with mean 0 and variance 1, then

2 $$\widehat {\tilde \beta } = cov({\mathbf{g}},{\tilde{\mathbf{y}}}) = cov({\mathbf{g}},r{\mathbf{y}} + {\mathbf{\epsilon }}) = cov({\mathbf{g}},r{\mathbf{y}}) + cov({\mathbf{g}},{\mathbf{\epsilon }}) = r\hat \beta$$

The standard association test statistics *x*_ground_ with high-coverage estimates is given by the following formula:

3 $$x_{\rm{ground}} = Ncor^2({\mathbf{g}},{\mathbf{y}}),$$

The association test statistics at low-coverage, *x*_low-coverage_ is

4 $$x_{\rm{low - coverage}} = Ncor^2({\mathbf{g}},\widetilde {\mathbf{y}}) = N\widehat {\tilde \beta }^2 = N(r\hat \beta )^2 = r^2 \cdot Ncor^2({\mathbf{g}},{\mathbf{y}})\, = \,\alpha \,x_{\rm{ground}}.$$

The quantity *αN* will be referred to as the effective sample size and denoted as *N*_eff_. For example, the same total sequencing budget can be distributed across 100 individuals yielding an effective sample size of 70 (*N* × *R*^2^ = 100 × 0.7 = 70) vs. 10 individuals at high-coverage for an effective sample size of 10 (*N* × *R*^2^ = 10 × 1 = 10).

Next, we investigated the properties of genes that are accurately quantified at low-coverage sequencing. Low-coverage sequencing expression estimates for highly expressed genes (mean cell-type-specific expression value (log-transformed TPM which is a standard preprocessing for single-cell data^37^) across individuals greater than 3) are highly correlated with the high-coverage (*R*^2^ ≈0.9–1.0, Fig. 1b). To exclude the inflation of *R*^2^ due to genes being expressed in only a small number of individuals, we assessed the accuracy of expression estimates for genes stratified by the number of individuals they are expressed in. Most genes are expressed in eight out of ten individuals (Supplementary Fig. 2a) and, although some genes are expressed only in one individual and their expression estimates tend to inflate the *R*^2^ (Supplementary Fig. 2b), their overall impact is negligible due to their small number.

To strengthen the evidence that cell-type-specific gene expression can be accurately inferred at low read coverage, we also analyzed a 10× dataset. Specifically, we downloaded the Census of Immune Cells^12^ dataset and considered a subset of it corresponding to bone marrow cells (we considered only lane 1, see “Methods”) consisting of 21,485 cells belonging to 8 donors. The initial read coverage of the dataset is approximately 20,000 reads per cell. We downsampled the reads at different levels of coverage (4000–20,000 reads per cell with step 2000) and computed cell-type-specific gene expression matrices. We restricted our analysis to the known 1341 marker genes encountered in bone marrow cell types^38^. We computed Pearson *R*^2^ between the low-coverage estimates and the high-coverage gene expression (i.e., the one computed at full coverage of 20,000 reads per cell). We observed that for example, at 4000 reads per cell, the average *R*^2^ in erythroblast cells is approximately 70% (Fig. 1c; see some example genes in Supplementary Fig. 3). The average *R*^2^ is high across all the genes (lowly and highly expressed, see Fig. 1d). Most genes are expressed in eight individuals (Supplementary Fig. 2c), and although some genes are expressed only in several individuals and their expression estimates tend to inflate the average *R*^2^ (Supplementary Fig. 2d), their overall impact is negligible due to their small number (similarly to the Smart-Seq2 dataset).

### Optimal power for ct-eQTL discovery is attained at lower coverage with larger number of individuals and cells

Having quantified the accuracy of cell-type-specific gene expression estimates at low-coverage sequencing, we next investigated the relationship between the statistical power for detecting eQTLs and effective sample size (“Methods”). Intuitively, as the number of reads per cell decreases, the accuracy of cell-type-specific gene expression estimates decreases due to sampling noise from sequencing and/or inaccurate cell-type identification. However, with lower coverage, many more individuals can be included in the study, thus increasing *N* for the same cost. To evaluate this relationship in realistic settings, which includes the number of cells per individual and sample preparation cost, we model the budget (in US dollars) as

5 $$B = B^m + \frac{{N \cdot L}}{x} + (10^{ - 6} \cdot p) \cdot N \cdot M \cdot r,$$

where *N* is the sample size, *M* is the target number of cells per individual (i.e, final number of measured cells), *r* is the read coverage, and *x* is the degree of sample multiplexing (number of individuals per reaction). *p* is the average cost of Illumina sequencing per 1 million reads (in US dollars), *L* is the library preparation cost per reaction (in US dollars), and *B*^*m*^ is the budget (in US dollars) wasted on sequencing of identifiable multiplets. *B*^*m*^ is an increasing nonlinear function of *N*, *M*, and *x* (for more details see “Methods”). Note that in the budget model of Eq. (5) we do not consider the details of the sequencing process (e.g., fixed flow-cell capacity) but let *p* account for that.

In what follows, we analyzed a 10× Genomics dataset (accession ID: GSE137029, see “Methods”). We selected a subset of this dataset consisting of 120 individuals each having at least 2750 cells (see “Methods”). We use (*N*, *M*, *r*) to refer to the experimental design. In all our experiments, the search space is defined by *N* ranging from 40 to 120 individuals in steps of 8 and *M* ranging from 500 to 2750 cells per individual in steps of 250. Specifically, for 120 individuals, if each pool contains 8 individuals, resulting in 15 pools, and the cost of library preparation per reaction is *L* = $2000, one needs to spend $30,000 for library preparation. In addition, when the average cost of Illumina sequencing per 1 million reads is set to *p* = $5, one needs to spend $5000 for sequencing 1 billion reads. With this amount of sequencing, when *N* = 120 and *M* = 2750, each cell is sequenced with *r* ≈3000 reads which is considered an extremely low coverage. Therefore, we fix the budget at *B* = $35,000.

**Fig. 2:**
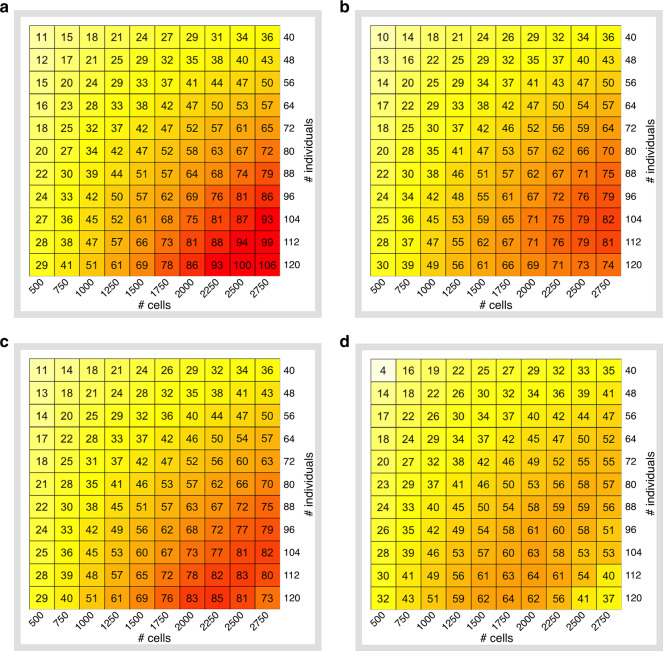
Effective sample size across a grid of experimental designs. Sample size *N* ranges from 40 to 120 individuals in steps of 8 and the number of cells per individuals *M* ranges from 500 to 2750 cells per individual in steps of 250 (CD4 T cells). **a** Library preparation is assumed to be 0$ per reaction, level of multiplexing is fixed and equal to 8. **b** Library preparation is set to $2000 per reaction, level of multiplexing is fixed and equal to 8. **c** Library preparation is set to $2000 per reaction, greedy multiplexing. **d** Library preparation is set to $2000 per reaction, greedy multiplexing, demultiplexing inaccuracy, and cell-type misclassification is taken into account.

First, when the sample preparation is $0/sample and each pool contains eight individuals (since according to demuxlet, 99% of the sample identities can be correctly recovered at this level of multiplexing^32^), we find that by sequencing all 2750 cells for all 120 individuals with a coverage of *r* = 14,500 reads per cell (note that *r* is greater than 3000 since in this case we assumed *L* to be 0) results in an *N*_eff_ of at least 102 for all cell types (Fig. 2a and Supplementary Fig. S4). This is in contrast with the standard strategy of *r* ≈50,000 reads per cell (Single Cell 3’ V2 chemistry, 10× Genomics^39^) which results in only 40 individuals under the same budget and *N*_eff_ = 36.

Next, we considered the impact of library preparation cost in designing a ct-eQTL study (Fig. 2b and Supplementary Fig. 5). At realistic costs of $2000/reaction, we find that the maximum *N*_eff_ over the search space which can be obtained with $35,000 is in the range of 67–86 across different cell types (Supplementary Fig. 5). The coverage in this case is 3000–5700 reads per cell. Note that the maximum effective sample size is not necessarily attained with 120 individuals and 2750 cells per individual. For example, for dendritic cells, sequencing 96 samples and 2750 cells per sample (at coverage *r* = 5700) yields *N*_eff_ = 67 which is better than with other experimental designs in the search space.

We also consider additional strategies for decreasing library preparation cost. A natural approach is to multiplex more individuals when possible (i.e., when *M* is not high). We refer to this approach as greedy multiplexing. We limit the per reaction capacity to 24,000 cells^30^ and allow *x* to take on the values up to 16 (see Fig. 2c and Supplementary Fig. 6). This will lower library preparation costs, but will increase the number of multiplets, i.e., droplets which contain at least two cells, and which are usually excluded from downstream analyses. In this scenario, the effective sample size can be increased considerably. For example, for dendritic cells, the experimental design (*N* = 120, *M* = 2000, *r* = 8000) yields *N*_eff_ = 70 vs. the case when only eight individuals can be multiplexed per reaction, which yields *N*_eff_ = 48. For a small budget, library preparation dominates the total cost, which limits how many individuals and cells can be sequenced. However, for a larger budget (≳$35,000), library preparation has less impact on the total cost due to multiplexing and the gain in power is incremental (compare Supplementary Figs. 5 and 6).

Next, we quantified how uncertainty in demultiplexing and cell-type identification at low-coverage affects our approach (see Fig. 2d and Supplementary Fig. 7). The aforementioned results clearly show that low-coverage sequencing is beneficial for increasing statistical power when cell types and sample labels for each cell are known. However, with an extremely low coverage, assigning a cell to the correct sample (demultiplexing) or cell type can be problematic which affects estimates of cell-type-specific gene expression and results in the loss of power.

**Fig. 3:**
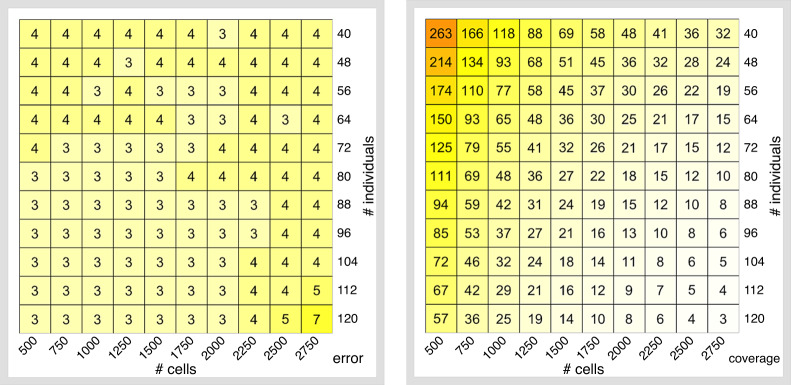
Cell-type misclassification error rate (%) and coverage (thousands of reads per cell). **a** Cell-type misclassification error; **b** coverage. Color scales correspond to the magnitude of the values in each cell of the heatmap.

To estimate demultiplexing accuracy at low coverage, we considered a subset of the 10× Genomics dataset (one reaction, 16 individuals). We then downsampled this dataset at different levels of coverage and ran demuxlet in order to compute *d* = *d*(*r*)—the proportion of cells which are assigned to the correct sample (Supplementary Fig. 8). Demultiplexing accuracy is high (~90%) at coverages ≥10,000 reads per cell. When coverage is below that value, demultiplexing accuracy declines rapidly. To account for demultiplexing inaccuracy at coverage *r*, before analyzing the experimental design (*N*,*M*,*r*), we first randomly permute sample labels for (1 − *d*(*r*))% of cells. To account for cell-type misclassification, we inferred cell type labels by label transfer using a reference PBMC dataset (instead of using the high-coverage labels, see “Methods”). Label transfer is a two-stage approach based on the paradigm of mutual nearest neighbor matching between a query and a reference dataset. In the first stage, a set of anchor query-reference pairs is identified. Anchors represent cell pairs with highly similar state cells across the datasets. The cell types of the cells in each pair are supposed to be the same. In the second stage, the prediction of the cell-type label of each non-anchor cell in the query dataset is performed based on an appropriate weighting scheme between each anchor and each non-anchor cell. Using this approach, the misclassification rate is approximately 4% across most of the experiments irrespectful of coverage (see Fig. 3a and Supplementary Fig. 9). Both assigning cells to the wrong sample and cell-type results in reduced power compared to having known cell-type labels. Nevertheless, at low coverages (at approximately 10,000 reads per cell), the effective sample size is still higher across all cell types (see Supplementary Figs. 10 and 11 for the coverage and the effective sample size). For any particular cell type (e.g., CD4 T cells), low-coverage sequencing delivers high levels of power irrespective of the budget which is allocated for the experiment (see Supplementary Fig. 11).

**Fig. 4:**
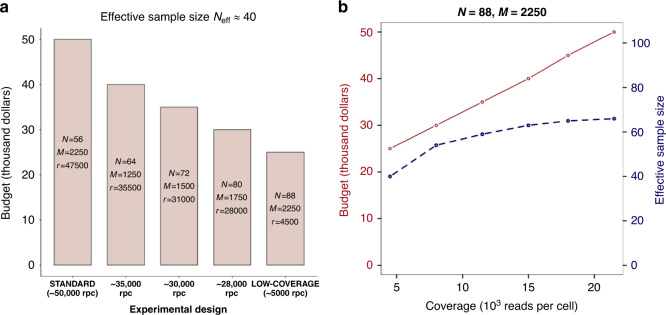
Experimental designs for CD4 T cells ct-eQTL with effective sample size *N*_eff_ = 40. **a** Comparison of different experimental designs. Experimental design *N* = 88, *M* = 2250, *r* = 4500 yields 2-fold reduction in cost than the standard design. **b** For a fixed sample size and number of cells per individual, increasing coverage implies increasing the effective sample size (i.e., power) only up to a point. There is little gain in power at coverages greater than 12,500 reads per cell. Red solid line corresponds to budget and blue dashed line corresponds to effective sample size.

To show the practical value of our approach, we compared different experimental designs for a fixed effective sample size. For example, *N*_eff_ = 40 for CD4 T cells can be attained by sequencing 56 individuals with a large number of cells at high coverage (in Fig. 4a, standard design). In this case, the total cost is $50,000. By increasing the sample size (and thus, decreasing the coverage), one can significantly reduce the cost of the experiment (Fig. 4a). With the low-coverage experimental design (4500 reads per cell), one obtains the same power in a ct-eQTL study with half of the budget ($25,000 vs. $50,000). Figure 4b shows that for a fixed sample size and number of cells per individual increasing coverage above ≈10,000 reads per cell causes only a small increase in effective sample size *N*_eff_ .

**Fig. 5:**
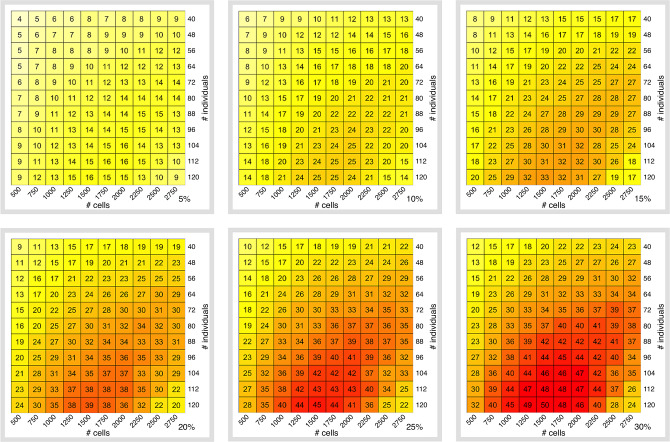
Effective sample size as a function of cell-type prevalence. Shown here is the effective sample size across the grid of experimental design when the cell-type abundance is set to different values −5, 10, 15, 20, 25, and 30%. (CD4 T cells at fixed budget $35,000). Color scales correspond to the magnitude of the values in each cell of the heatmap.

We next analyzed the impact of cell-type frequency on the power in a ct-eQTL study. In general, ct-eQTL studies in a more frequent cell type (for example, CD14+ monocytes and CD4 T cells) have higher power (Supplementary Fig. 12). The adjusted Pearson *R*^2^ between the cell counts and the effective sample size is high (for example, when *B* = $35,000, *N* = 96 individuals, *M* = 2000 cells per individual, and *r* = 12,500 reads per cell, it is equal to 0.72 (Supplementary Fig. 13)). Despite the fact that the effective sample size values are different for different cell types across different budgets, the optimal coverage (i.e., coverage, at which we observe the maximum effective sample size across the explored experimental designs) is 10,000 ± 2500 reads per cell (Supplementary Fig. 14). To study how effective sample size depends on cell-type prevalence, we simulated datasets with different levels of CD4 T cells prevalence ranging from 5 to 30% (Fig. 5). As expected, for one particular cell type, effective sample size is a monotonic function of prevalence (given all other parameters are fixed). Nevertheless, the highest effective sample size is achieved at coverages around 10,000 reads per cell irrespective of cell-type prevalence (Supplementary Fig. 15).

Finally, we sought to determine whether imputation tools for scRNA-Seq can increase the effective sample size of ct-eQTL studies. If this would be the case, one could use imputation tools to reduce the number of required cells per sample. We used scImpute^40^ to improve the quality of the dataset with *N* = 120, *M* = 2750, and *r* = 2000 reads per cell at budget *B* = $35,000 (the experimental design with the lowest considered coverage) and observed no improvement in the effective sample size. For example, for CD4 T cells, we observed *N*_eff_ = 37 which is equal to the effective sample size for this experimental design without using scImpute (Supplementary Fig. 11).

### Cell-type eQTL power analysis in empirical data

For the budget *B* = $35,000, we performed ct-eQTL analyses for each experimental design (*N*, *M*, *r*), where *N* ranged from 40 to 120 with step 8 and *M* ranged from 500 to 2750 with step 250. We also ran the ct-eQTL analysis on the original dataset to obtain the “high-coverage” set of ct-eQTLs. We considered the following accuracy metrics:

1. Recall—the percentage of high-coverage ct-eQTLs recovered in the experiment. It is an empirical estimate of the statistical power.
2. Precision—the percentage of high-coverage ct-eQTLs among all the ones called in the experiment.

Supplementary Figure 16a shows an upward trend in the estimate of statistical power as the sample size grows. Due to sampling variance in our experiments (when sampling individuals and cells from the full dataset), we do observe some variance along the fitted line. Despite this fact, an experiment with a higher effective sample size leads to higher statistical power to detect true associations. Clearly, low-coverage experimental designs (with coverage less than 50,000 reads per cell) yield higher estimates of power than the high-coverage ones.

**Fig. 6:**
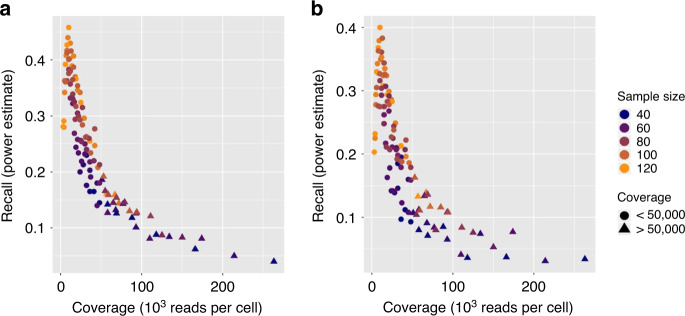
Performance of ct-eQTL analysis. Shown here is recall (power estimate) as a function of coverage in the ct-eQTL analysis of CD4 T cells at fixed budget $35,000. **a** Mean ct-eQTL; **b** variance ct-eQTL.

The power (and, consequently, the number of discovered high-coverage ct-eQTLs) inversely depends on the coverage (Fig. 6). For a fixed number of individuals, the highest power is achieved at the lowest coverage. This means that for a ct-eQTL analysis, the best strategy under a fixed budget is to spread the reads across many individuals. On average, the optimal design with low-coverage sequencing yields three times more power than the designs using current standard level of coverage (50,000 reads per cell). Notably, low-coverage sequencing yields a high level of precision—percentage of the high-coverage ct-eQTLs in the output of the analysis (Supplementary Fig. 16b).

In addition, we explored cell-type specific variance eQTL analysis as opposed to cell-type specific mean eQTL. We restricted our analysis to CD4 T cells (the most abundant cell type) at *B* = $35,000. For this level of budget, similar to the above analysis of mean ct-eQTL, we performed variance ct-eQTL analyses for each experimental design (*N*, *M*, *r*), where *N* ranged from 40 to 120 with step 8 and *M* ranged from 500 to 2750 with step 250. We also ran the variance ct-eQTL analysis on the original dataset to obtain the high-coverage set of variance ct-eQTLs. As in the case of mean eQTL, low-coverage experiments yield higher power for variance eQTL analysis (Fig. 6b).

## Discussion

In this work, we show that cell-type-specific gene expression can be inferred with low-coverage sequencing given a sufficient number of cells per individual per cell type. Leveraging this fact, we used the largest available scRNA-Seq dataset (with respect to sample size, *N* = 120) in order to quantify the impact of the number of reads, number of individuals, number of cells, level of sample multiplexing, and cell-type classification accuracy on the power of ct-eQTL studies. We recommend that for a highly efficient ct-eQTL study, one should increase the sample size and the number of cells per sample while keeping the coverage at 10,000 reads per cell in order to achieve better statistical power without increasing budget.

We conclude with several caveats and future directions. First, at low-coverage sequencing, poor cell-type identification can lead to the inability to detect some rare cell types. In our approach, we use label transfer from a PBMC reference dataset which is well-studied and for which the cell-type labels are reliable. However, in case of missing reference dataset cell-type identification can be less accurate and, thus, negatively affect statistical power. In case when a reference dataset is available, one should use the label transfer procedure to accurately annotate cells with cell-type labels. However, even at 10,000 reads per cell, one can reliably identify cell types without any reference dataset^10^.

Second, the budget model used in our paper assumes that sequencing cost per one million reads is a fixed parameter *p*. However, this assumption hides the underlying details of sequencing such as, for example, flow cells (S2, NextSeq, etc.) used in the experiments. Flow cells have different output capacities, and therefore, the number of flow cells used in the experiment may vary, affecting the average costs per one million reads. For a more fine-grained budget modeling of scRNA-Seq, one has to account for such details and, therefore, use a more sophisticated (and more realistic) budget model.

Third, our simulations of read counts at low coverage (multinomial sampling) provide only the results for an average case scenario, i.e., we assume that the coverage is uniform. However, in practice, coverage can be biased, meaning that in low-coverage scenarios, more UMIs can be unobserved than we detect in simulations.

Fourth, the numbers of variance QTLs that we found in this study do not agree with the conclusions of a recent study that large-sample sizes (over 4000) are required to have reasonable power to detect variance QTLs^41^. There are three reasons for the difference between our results and ref. ^41^. First, Sarkar et al.^41^ used a very small single-cell RNA-Seq dataset. Their dataset consists of 53 individuals with 5447 single cells in total. Therefore, their estimates of the mean and variance of the gene expression for each individual should be extremely noisy. Second, that paper considers a different cell type (iPSC cells). Third, a different model for variance QTL analysis was used in ref. ^41^. As opposed to their approach, we considered the variance computed from log-transformed total-UMI-count-scaled UMI counts. We also used ~3 times more variants for the eQTL analyses as compared with ref. ^41^ (21.4 vs 8.4 million of variants).

Finally, we considered sample multiplexing of at most 16 individuals per reaction. However, one could explore even higher levels of multiplexing. With multiple samples per reaction, demultiplexing may be challenging. But if the samples are genetically heterogeneous, demultiplexing is almost error-free, meaning that more samples can be sequenced in the experiment.

## Methods

### Budget model

We assume a fixed budget *B* = *B*^*L*^ + *B*^*S*^ + *B*^*m*^, where *B*^*L*^ is the cost of the library preparation, *B*^*S*^ is the cost of sequencing, and *B*^*m*^ is the extra cost due to non-identifiable multiplets which are discarded in the downstream analysis. For 10× Genomics, *B*^*L*^ >> *B*^*S*^. Recent advances in single-cell computational methods^32^ allow to accurately demultiplex cells of individuals with a variable genetic background which were pooled in one reaction. This considerably reduces the library preparation costs. However, multiplexing usually results in overloading of the sequencing instrument, which increases the number of multiplets. Identifiable multiplets are excluded from downstream analysis. However, the multiplets that cannot be identified remain in the dataset. The amount of money spent on sequencing of identifiable multiplets *B*^*m*^, increases with the number of cells per reaction. When conducting an scRNA-Seq experiment, we must decide the number of individuals *N* and the number *M* of cells per individual to be sequenced. Based on these two parameters, we can determine *r*, the number of reads per cell. Assuming that the library preparation cost per reaction is *L*, our model for the budget is

6 $$B = B^m + \frac{{N \cdot L}}{x} + (10^{ - 6} \cdot p) \cdot N \cdot M \cdot r,$$

7 $$B^m = f(N,M,x),$$

where *x* is the number of individuals per 10× reaction (sample multiplexing), and *f* is a function of sample size, number of cells per individual and the level of multiplexing to the budget spent on sequencing the multiplets. The function *f* is nonlinear and it is increasing in all three parameters. The function is implemented based on the code of the Satija lab single-cell cost calculator (https://satijalab.org/costpercell).

To get an estimate of the number of reads per cell *r* in a scRNA-Seq experiment with N individuals and *M* cells per individual, we do the following:

1. Compute the budget for the sequencing itself for one reaction: $B_S = \frac{x}{N} \cdot \left( {B - \frac{{N \cdot L}}{x}} \right)$.
2. For the computed value of *B*_*S*_ and the number of cells in a batch (which is equal to *M*·*x*), find the number of reads *r* which can be requested for sequencing. We use the computational model for the cost described in the Satija lab single-cell cost calculator. Our heuristic uses a dichotomy search to determine the actual number of reads per cell which we can obtain with the given sequencing budget *B*_*S*_, the given values of cells per reaction, number of multiplexed samples, and other experimental details.

The whole workflow is described in Supplementary Fig. 17.

### Read count simulations for 10× Genomics

Simulating low-coverage experiments for single-cell RNA-Seq data should be performed by downsampling reads. However, this might not be feasible from a computational point of view. The large amount of data (several Terabytes) as well as the processing time represent a bottleneck. To overcome this issue, we propose the following approach for simulating low-coverage datasets from a larger dataset represented by a gene-UMI count matrix *X*. First, we assume that the values in *X* reflect the true gene expression, i.e., *X*_*ij*_ is the number of transcripts produced by the gene *i* in cell *j*. Second, we assume that each cell’s transcriptome is sequenced with approximately the same number of Illumina reads *r*. Third, we assume that the number of reads per transcript is approximately the same. Then, to simulate the number of Illumina reads sequenced from each UMI of a cell *j* given that the total of *r* Illumina reads were sequenced, we draw them from the following multinomial distribution

8 $$R_j^r \sim Multinomial\left( {r,\frac{1}{{S_j}}(1,1,...,1)} \right),$$

where $S_j = \mathop {\sum}\nolimits_1^m {X_{ij}^r}$ is the total number of UMIs in cell *j* and the length of the vector $R_j^r$ is equal to the total number of UMIs in the cell *j* when *r* is small, some of the UMIs can drop out (meaning that they were not captured by the in-silico sequencing procedure) and as a result, the observed UMI counts for the gene *i* can become smaller. When *r* is large, there will be a saturation point after which increasing the read coverage will not improve the gene expression estimates (see Supplementary Fig. 18). For each gene, we count the number of non-zero values at the corresponding positions in $R_j^r$ and set it as the simulated UMI count for the gene *i* in the cell *j* (see Supplementary Fig. 19).

### Datasets

We used a 10× Genomics dataset consisting of 142 genotyped individuals with the number of cells ranging from 2000 to 8000 per individual (accession ID: “GSE137029”). The dataset has undergone the standard analysis using the 10× Cell Ranger (version 3.0.2) software package. For our analysis, we selected only the individuals with at least 2750 cells and downsampled the number of cells for each individual to this value. Thus, we obtained a dataset of 120 individuals, each having 2750 cells. Eight cell types were present in the dataset: B cells, CD14+ monocytes, CD4 T cells, CD8 T cells, dendritic cells, FCGR3A+ monocytes, megakaryocytes, and NK cells (Supplementary Fig. 20). The individuals were genotyped on the Affymetrix World LAT Array. The raw intensity .CEL files were processed according to the APT best practices using the Analysis Power Tools command line utility (APT). Poor quality probe sets were removed using APT’s SNPpolisher functionality. Resulting genotypes were used for imputation with the Haplotype Reference Consortium version 1.1 and a MAF < 0.01. The genotypes were imputed using Minimac3. The total of 21,412,068 variants were used in the analysis.

### Cell-type classification

Cell types were determined by using label transfer feature from Seurat (version 3.0) using the default parameters^42^. As the reference, the 2700 PBMC 10× dataset was used^43^. For imputation, scImpute (version 0.0.9) with cell-type labels inferred by label transfer and the rest of parameters set to their default values was used.

### Cell-type-specific expression profiles

Computing cell-type-specific expression profiles was done by grouping the cells based on their cell types and then aggregating the UMI counts (or TPMs) across the individuals for every gene. The aggregated expression profiles were scaled by 1 million and log-normalized.

### Mean and variance ct-eQTL analysis

For the mean and variance ct-eQTL and analysis, we used MatrixEQTL^44^ R package. We performed cis-ct-eQTL mapping with cis-distance set to 1 Mbp and *p* value threshold set to 5% (Student’s *t* test). As covariates, we used race and disease status. For the variance ct-eQTL analysis, we also used the mean expression as a covariate to account for the mean-variance relationship. The resulting SNP-gene pairs were filtered at an FDR threshold of 5%. The genomic coordinates for each gene were obtained from the GRCh38 genomic annotations downloaded from the Ensemble (release 94).

### Reporting summary

Further information on research design is available in the Nature Research Reporting Summary linked to this article.

## Supplementary information

Supplementary Information

Peer Review File

Reporting Summary

## Data Availability

The Smart-Seq2 dataset is publicly available on ArrayExpress (EBI) under the accession number “E-MTAB-5061”. 10X Genomics Census of Immune Cells dataset was downloaded from the “Human Cell Atlas Data Portal[https://data.humancellatlas.org/explore/projects/cc95ff89-2e68-4a08-a234-480eca21ce79]”. 10X dataset used in the simulations are publicly available on Gene Expression Omnibus (GEO) under the accession number “GSE137029”.

## Code availability

The scripts for reproducing the results of the paper are available online: https://github.com/mandricigor/ct-eqtl-design. The online calculator for the design of ct-eQTL studies is available at https://mandricigor.github.io/ct-eqtl-design/.
