# Supplementary material for: Optimized design of single-cell RNA sequencing experiments for cell-type-specific eQTL analysis
Source: Nat Commun. 2020 Oct 30;11:5504. doi: 10.1038/s41467-020-19365-w (PMC7599215; doi:10.1038/s41467-020-19365-w)
Supplement: Supplementary file 1 — Supplementary Information [file 41467_2020_19365_MOESM1_ESM.pdf]

Supplementary Information

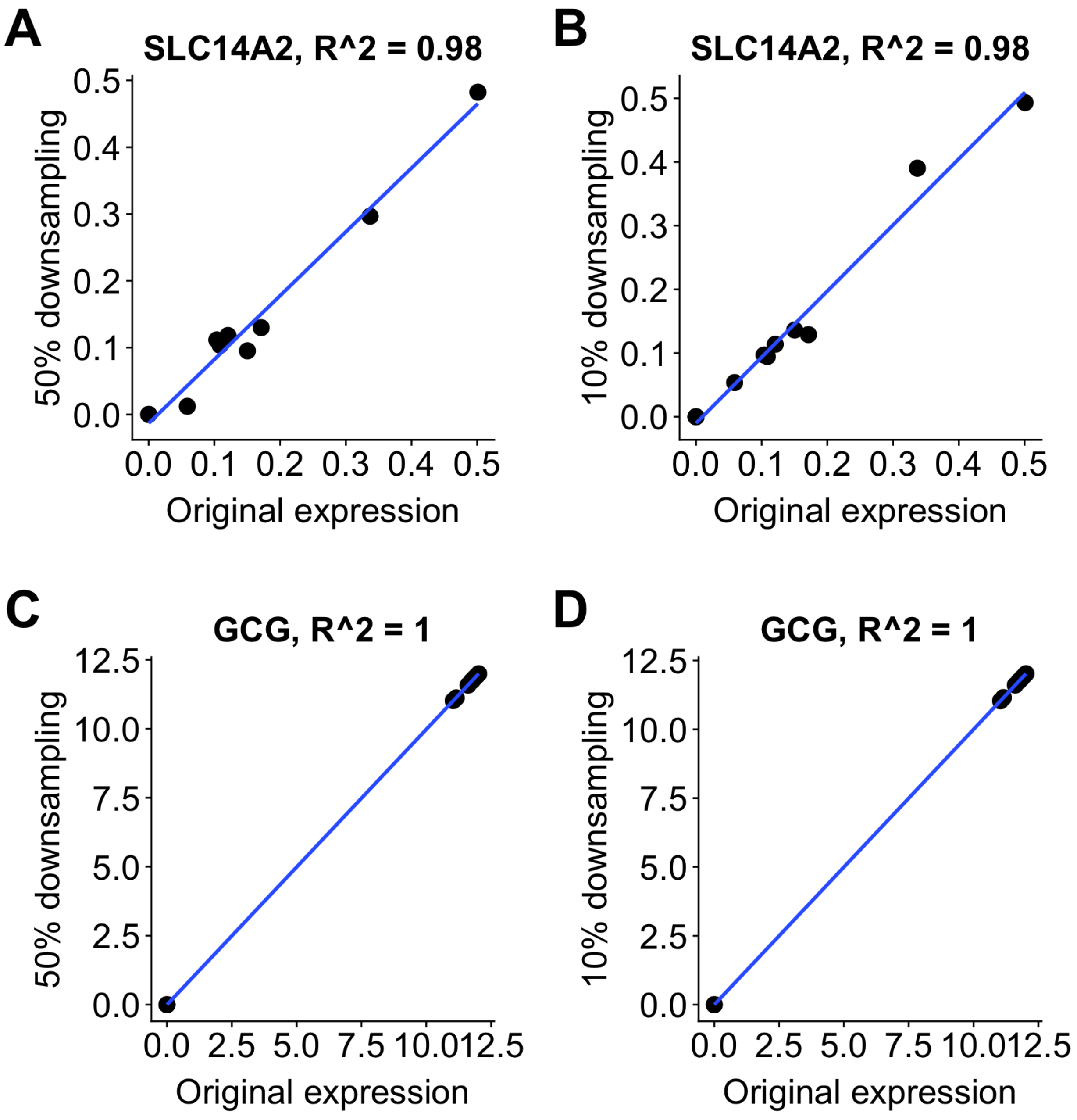

Supplementary Figure 1: Pearson R^2 between low-coverage estimates and the high-coverage gene expression in Smart-Seq2 dataset. A) *SLC14A2* gene, 50% downsampling (≈ 375,000 reads per cell); B) *SLC14A2* gene, 10% downsampling (≈ 75,000 reads per cell); C) *GCG* gene, 50% downsampling (≈ 375,000 reads per cell); D) *GCG* gene, 10% downsampling (≈ 75,000 reads per cell).

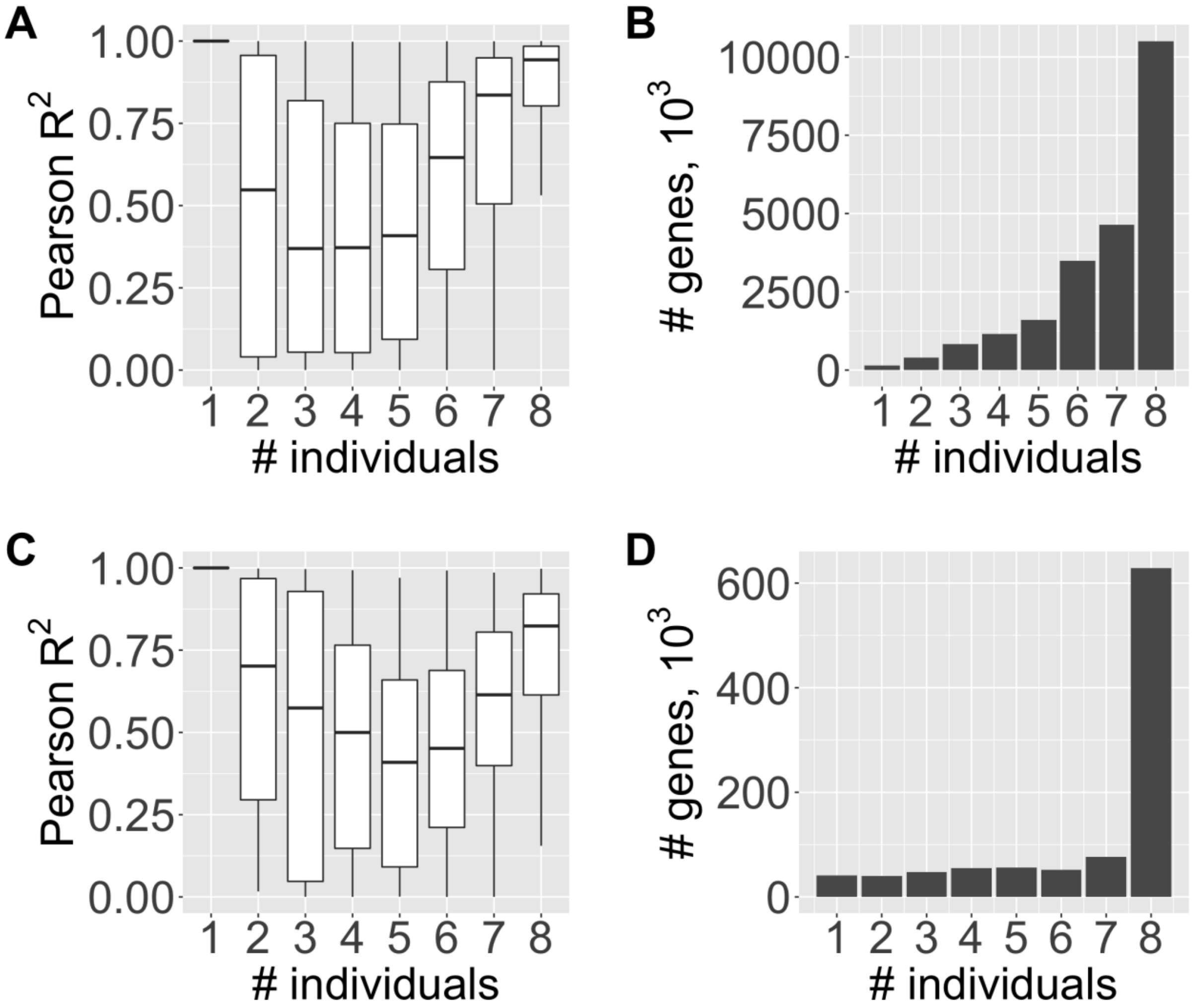

Supplementary Figure 2: Stratification of genes based on the number of individuals they are expressed in (Smart-Seq2 dataset). A) Distribution of genes by the number of individuals they are expressed in, Smart-Seq2 dataset. B) Average Pearson R^2 at 75,000 reads per cell (alpha cells) stratified by the number of individuals they are expressed in, Smart-Seq2 dataset (vertical bars indicate interquartile range). C) Distribution of genes by the number of individuals they are expressed in, 10X dataset (The Census of Immune Cells). D) Average Pearson R^2 at 4,000 reads per cell (erythroblast cells) stratified by the number of individuals they are expressed in, 10X dataset (The Census of Immune Cells) (vertical bars indicate interquartile range).

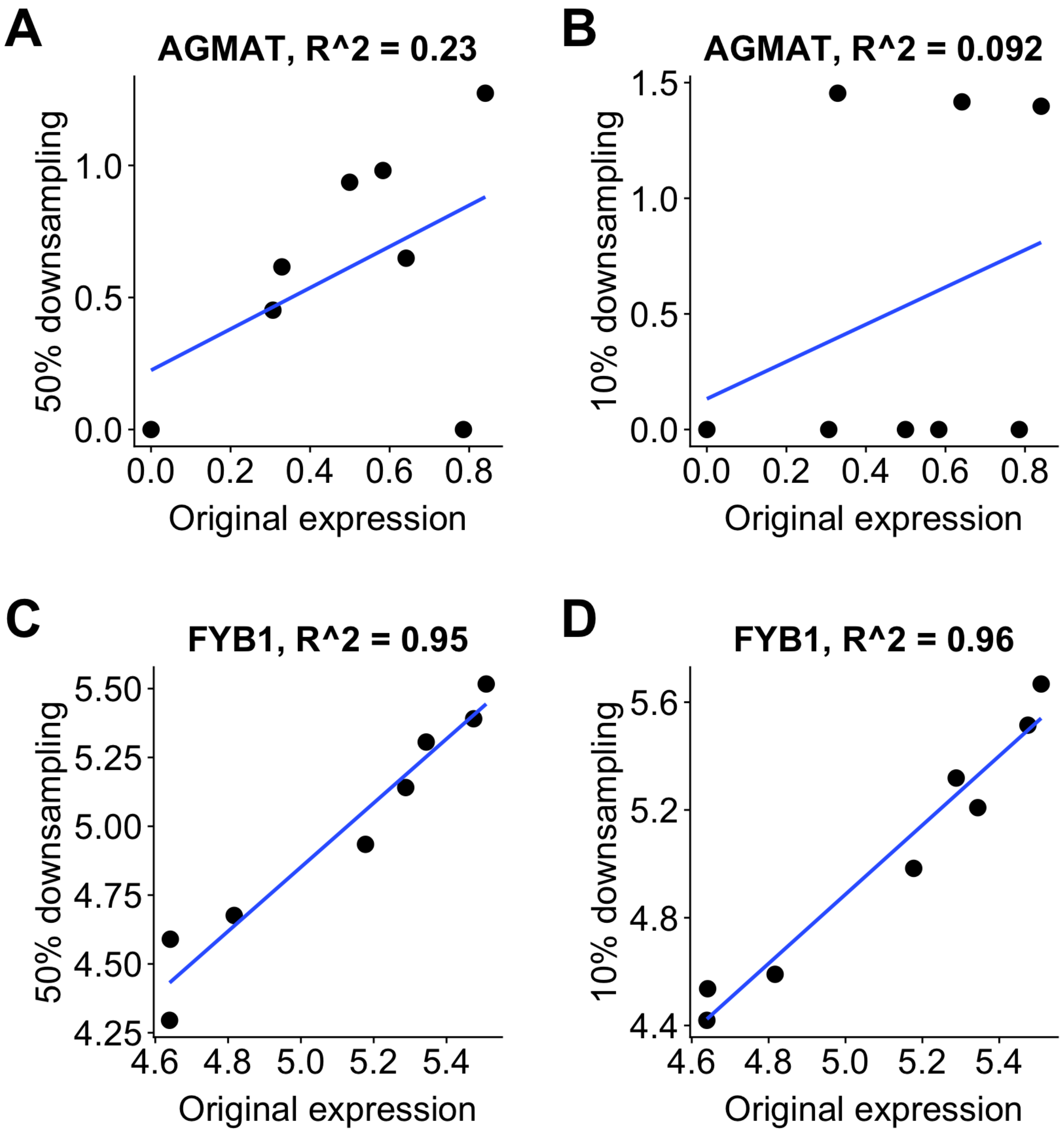

Supplementary Figure 3: Pearson R^2 between low-coverage estimates and the high-coverage gene expression in a 10X dataset (subset of the Census of Immune cells). A) *AGMAT* gene, 50% downsampling (≈ 10,000 reads per cell); B) *AGMAT* gene, 10% downsampling (≈ 2,000 reads per cell); C) *FYB1* gene, 50% downsampling (≈ 10,000 reads per cell); D) *FYB1* gene, 10% downsampling (≈ 2,000 reads per cell).

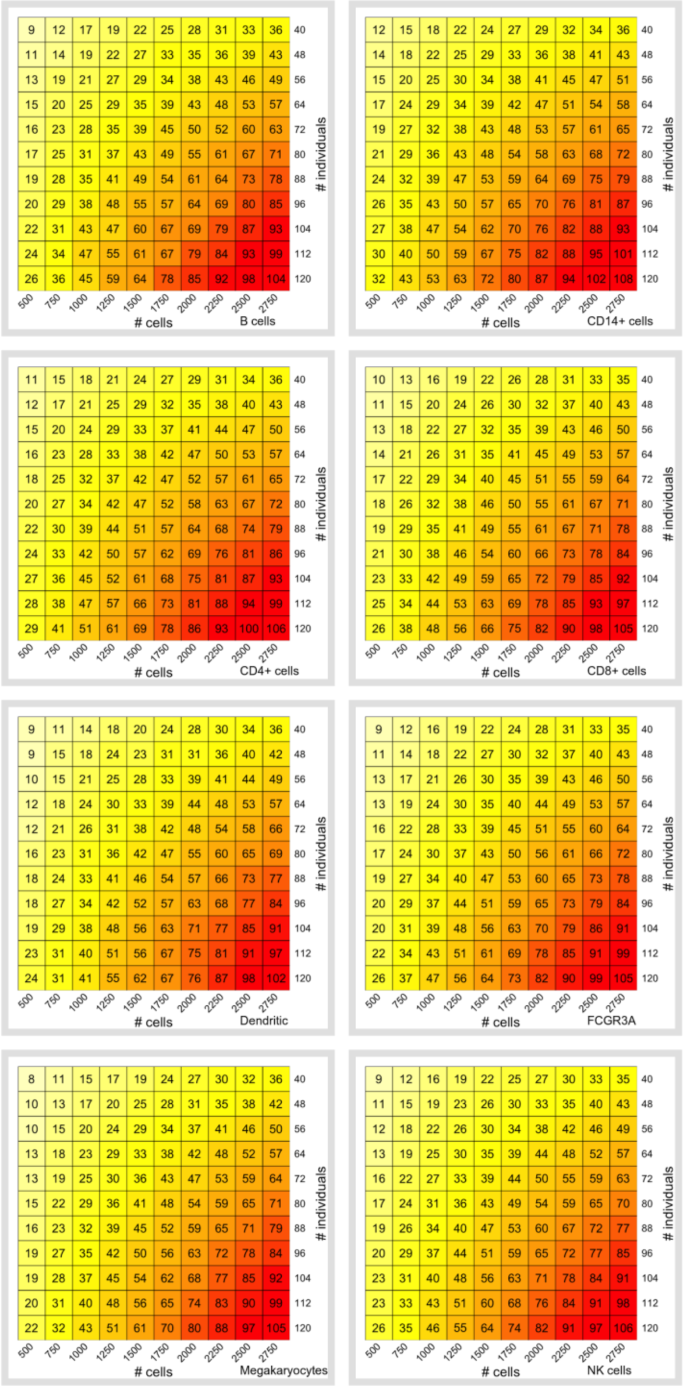

Supplementary Figure 4: Effective sample size as a function of number of individuals and number of cells per individual at budget \$35,000 assuming no library preparation cost, multiplexing of 8 individuals per reaction, and known cell types. The dependence on read coverage is implicit. The maximum effective size N_{eff} is 105 for B cells ($N = 120, M = 2{,}750, r = 14{,}500$), 108 for CD14+ cells ($N = 120, M = 2{,}750, r = 14{,}500$), 107 for CD4+ cells ($N = 120, M = 2{,}750, r = 14{,}500$), 105 for CD8+ cells ($N = 120, M = 2{,}750, r = 14{,}500$), 102 for dendritic cells ($N = 120, M = 2{,}750, r = 14{,}500$), 106 for Fcgr3a cells ($N = 120, M = 2{,}750, r = 14{,}500$), 105 for megakaryocytes ($N = 120, M = 2{,}750, r = 14{,}500$), 106 for NK cells ($N = 120, M = 2{,}750, r = 14{,}500$). Colour scales correspond to the magnitude of the values in each cell of the heatmap.

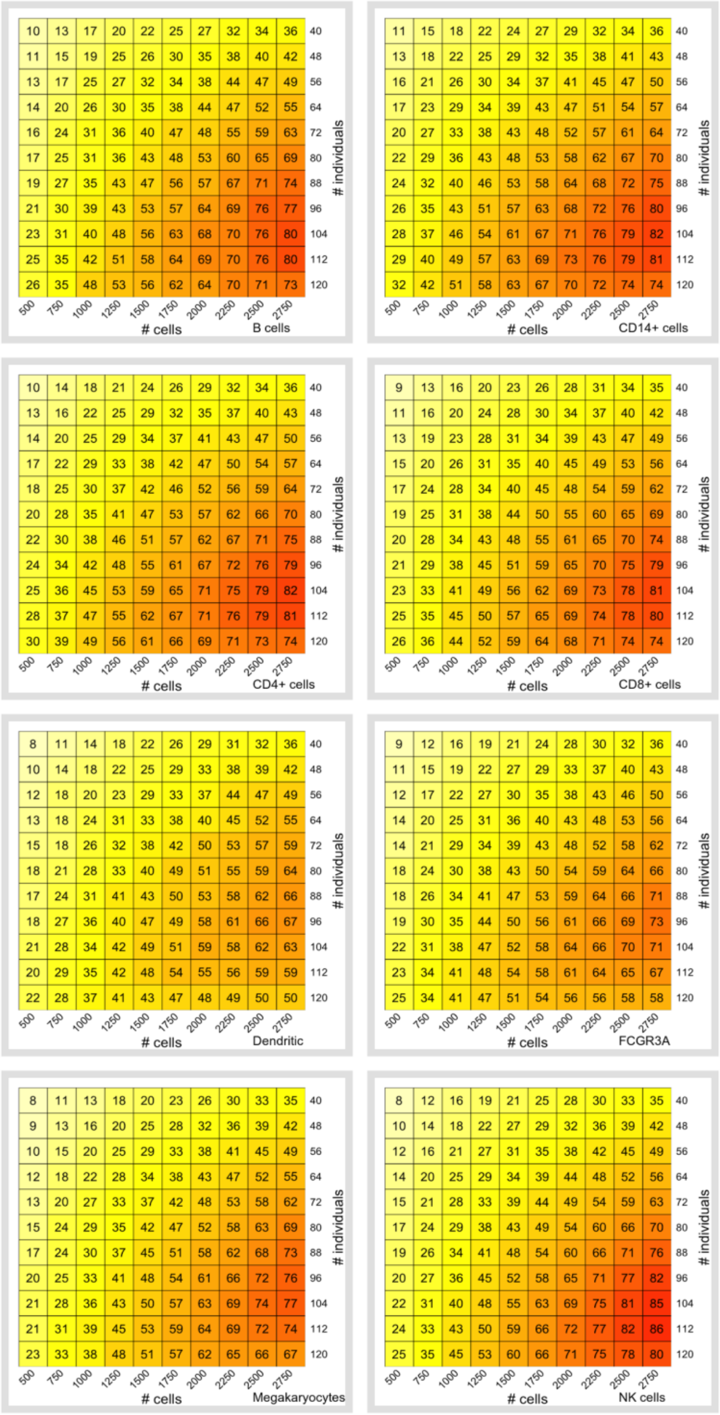

Supplementary Figure 5: Effective sample size as a function of number of individuals and number of cells per individual at budget $35,000 assuming library preparation costs of $2,000 per reaction, multiplexing of 8 individuals per reaction, known cell types. The maximum effective size N_{eff} is 81 for B cells ($N = 104, M = 2{,}750, r = 4{,}300$), 82 for CD14+ cells ($N = 104, M = 2{,}750, r = 4{,}300$), 82 for CD4+ cells ($N = 104, M = 2{,}750, r = 4{,}300$), 82 for CD8+ cells ($N = 104, M = 2{,}750, r = 4{,}300$), 67 for dendritic cells ($N = 96, M = 2{,}750, r = 5{,}700$), 73 for Fcgr3a cells ($N = 96, M = 2{,}750, r = 5{,}700$), 77 for megakaryocytes ($N = 104, M = 2{,}750, r = 4{,}300$), 86 for NK cells ($N = 112, M = 2{,}750, r = 3{,}100$). Colour scales correspond to the magnitude of the values in each cell of the heatmap.

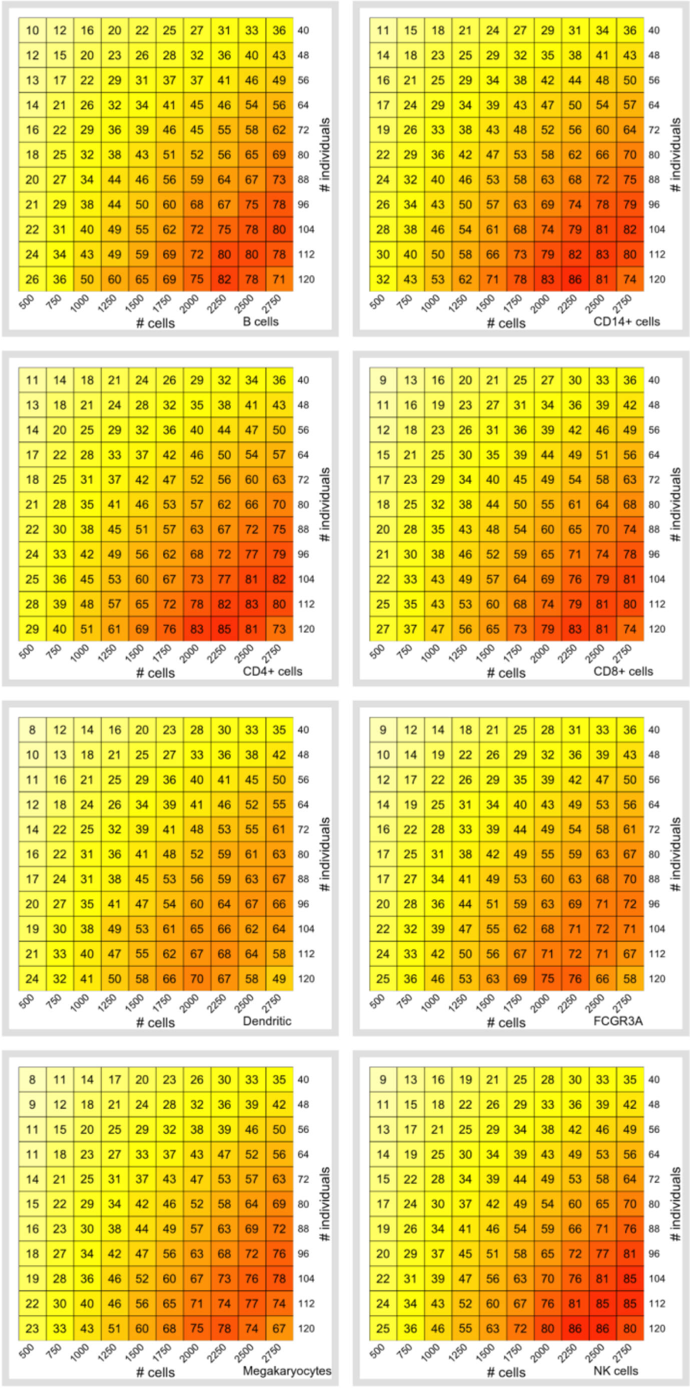

Supplementary Figure 6: Effective sample size as a function of number of individuals and number of cells per individual at budget $35,000 assuming library preparation costs of $2,000 per reaction, greedy multiplexing (up to 16 individuals per reaction) and known cell types. The level of multiplexing takes on values from 8 to 16. The maximum effective size N_{eff} is 83 for B cells ($N = 120, M = 2{,}250, r = 5{,}400$), 86 for CD14+ cells ($N = 120, M = 2{,}250, r = 5{,}400$), 86 for CD4+ cells ($N = 120, M = 2{,}250, r = 5{,}400$), 83 for CD8+ cells ($N = 120, M = 2{,}250, r = 5{,}400$), 70 for dendritic cells ($N = 120, M = 2{,}000, r = 8{,}000$), 76 for Fcgr3a cells ($N = 120, M = 2{,}250, r = 5{,}400$), 79 for megakaryocytes ($N = 120, M = 2{,}250, r = 5{,}400$), 86 for NK cells ($N = 120, M = 2{,}250, r = 5{,}400$). Colour scales correspond to the magnitude of the values in each cell of the heatmap.

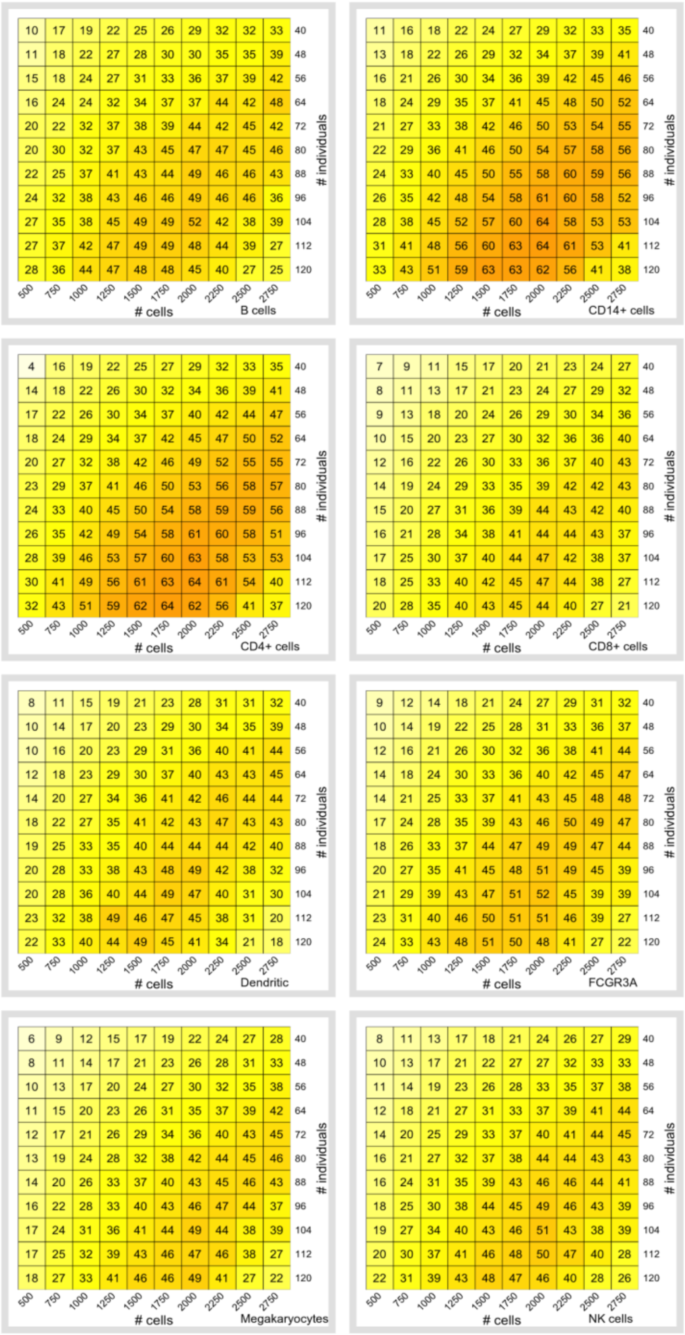

Supplementary Figure 7: Effective sample size as a function of number of individuals and number of cells per individual at budget $35,000 assuming library preparation costs of $2,000 per reaction, greedy multiplexing (up to 16 individuals per reaction) and unknown cell types. Demultiplexing inaccuracy at low coverage is taken into account. Cell types are inferred using Seurat's label transfer procedure. The maximum effective size N_{eff} is 52 for B cells ($N = 104, M = 2{,}000, r = 10{,}500$), 64 for CD14+ cells ($N = 112, M = 2{,}000, r = 8{,}500$), 64 for CD4+ cells ($N = 120, M = 1{,}750, r = 9{,}500$), 47 for CD8+ cells ($N = 112, M = 2{,}000, r = 8{,}500$), 49 for dendritic cells ($N = 96, M = 2{,}000, r = 12{,}500$), 52 for Fcgr3a cells ($N = 104, M = 2{,}000, r = 10{,}500$), 49 for megakaryocytes ($N = 120, M = 2{,}000, r = 7{,}500$), 51 for NK cells ($N = 104, M = 2{,}000, r = 10{,}500$). Colour scales correspond to the magnitude of the values in each cell of the heatmap.

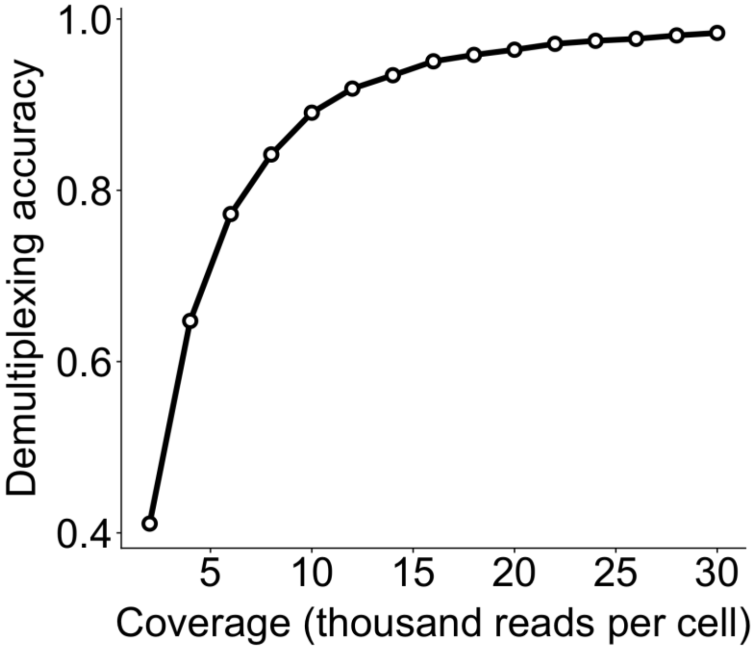

Supplementary Figure 8: The impact of read coverage on the accuracy of demultiplexing (10X dataset). Shown here is the percentage of correctly assigned cells (to the corresponding sample) as a function of average number of reads per cell.

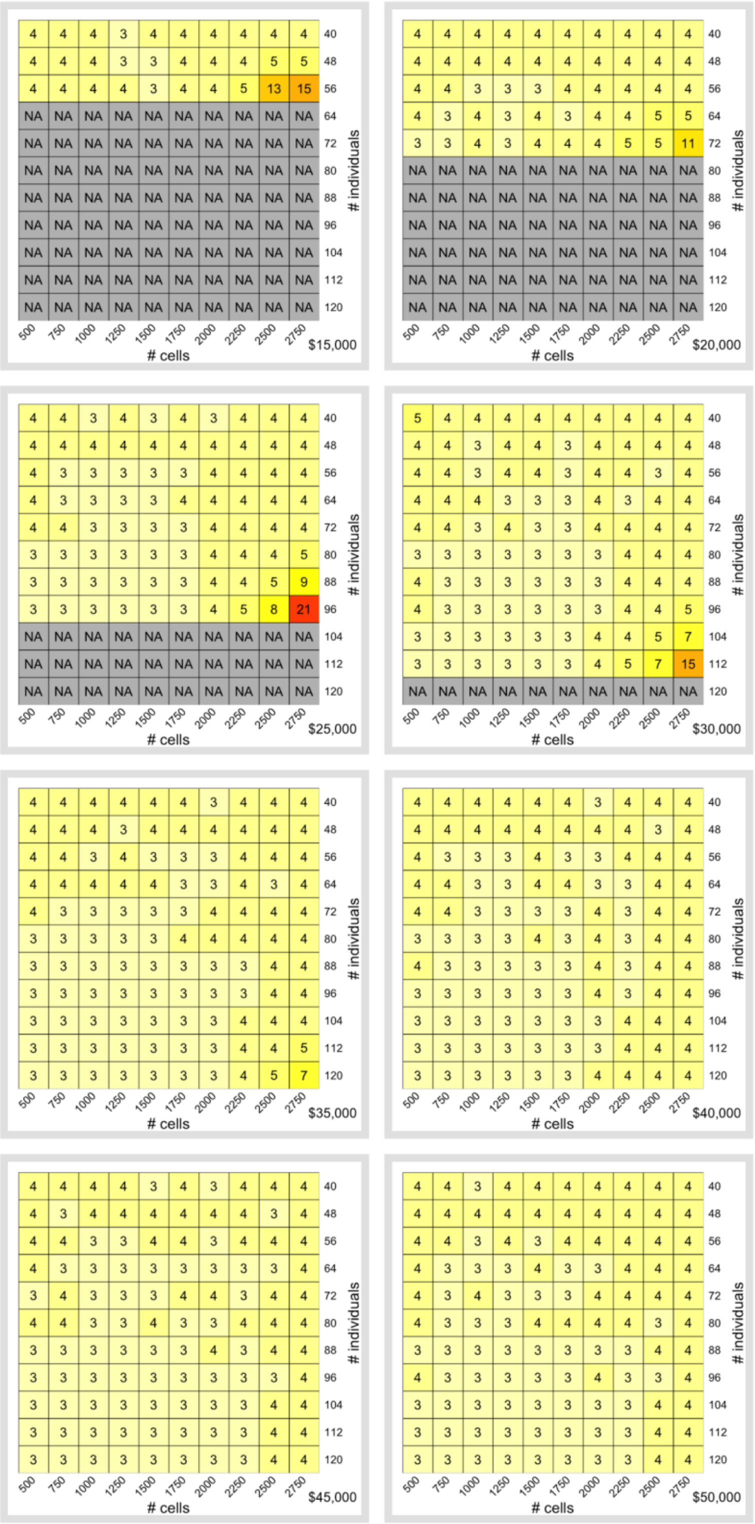

Supplementary Figure 9: Cell-type misclassification rate (in %) across different budgets. Library preparation costs of $2,000 per reaction and greedy multiplexing are assumed. Demultiplexing inaccuracy at low coverage is taken into account. Colour scales correspond to the magnitude of the values in each cell of the heatmap.

$15,000

# individuals \ # cells	500	750	1000	1250	1500	1750	2000	2250	2500	2750
40	82	52	37	28	22	15	13	11	8	7
48	67	42	29	21	16	12	10	7	4	4
56	46	29	20	15	12	8	6	4	1	1
64	NA	NA	NA	NA	NA	NA	NA	NA	NA	NA
72	NA	NA	NA	NA	NA	NA	NA	NA	NA	NA
80	NA	NA	NA	NA	NA	NA	NA	NA	NA	NA
88	NA	NA	NA	NA	NA	NA	NA	NA	NA	NA
96	NA	NA	NA	NA	NA	NA	NA	NA	NA	NA
104	NA	NA	NA	NA	NA	NA	NA	NA	NA	NA
112	NA	NA	NA	NA	NA	NA	NA	NA	NA	NA
120	NA	NA	NA	NA	NA	NA	NA	NA	NA	NA

$20,000

# individuals \ # cells	500	750	1000	1250	1500	1750	2000	2250	2500	2750
40	127	81	57	43	33	26	22	18	15	13
48	104	65	45	33	25	20	16	13	10	9
56	78	49	35	26	20	15	12	9	7	6
64	67	42	29	21	16	12	9	6	4	4
72	50	32	22	17	13	9	8	4	3	2
80	NA	NA	NA	NA	NA	NA	NA	NA	NA	NA
88	NA	NA	NA	NA	NA	NA	NA	NA	NA	NA
96	NA	NA	NA	NA	NA	NA	NA	NA	NA	NA
104	NA	NA	NA	NA	NA	NA	NA	NA	NA	NA
112	NA	NA	NA	NA	NA	NA	NA	NA	NA	NA
120	NA	NA	NA	NA	NA	NA	NA	NA	NA	NA

$25,000

# individuals \ # cells	500	750	1000	1250	1500	1750	2000	2250	2500	2750
40	173	109	77	58	45	37	31	26	22	19
48	140	88	61	45	34	28	23	19	16	14
56	110	69	49	37	28	22	18	15	12	10
64	94	59	41	30	23	18	14	11	9	7
72	75	47	33	25	19	15	12	8	7	5
80	67	42	29	21	16	12	10	7	5	4
88	53	33	24	18	14	10	7	5	4	2
96	48	30	21	16	12	8	6	4	2	1
104	NA	NA	NA	NA	NA	NA	NA	NA	NA	NA
112	NA	NA	NA	NA	NA	NA	NA	NA	NA	NA
120	NA	NA	NA	NA	NA	NA	NA	NA	NA	NA

$30,000

# individuals \ # cells	500	750	1000	1250	1500	1750	2000	2250	2500	2750
40	218	138	97	73	57	48	40	33	29	25
48	178	111	77	57	43	37	30	26	22	19
56	142	89	63	47	36	30	24	20	17	15
64	122	76	53	39	29	24	20	16	13	11
72	100	63	44	33	25	20	16	13	11	9
80	89	56	39	29	22	17	14	11	9	7
88	74	46	33	24	19	14	11	9	7	5
96	67	42	29	21	16	12	10	7	5	4
104	55	35	24	18	14	11	8	5	4	2
112	51	32	22	16	12	9	6	4	2	1
120	NA	NA	NA	NA	NA	NA	NA	NA	NA	NA

$35,000

# individuals \ # cells	500	750	1000	1250	1500	1750	2000	2250	2500	2750
40	263	166	118	88	69	58	48	41	36	32
48	214	134	93	68	51	45	36	32	28	24
56	174	110	77	58	45	37	30	26	22	19
64	150	93	65	48	36	30	25	21	17	15
72	125	79	55	41	32	26	21	17	15	12
80	111	69	48	36	27	22	18	15	12	10
88	94	59	42	31	24	19	15	12	10	8
96	85	53	37	27	21	16	13	10	8	6
104	72	46	32	24	18	14	11	8	6	5
112	67	42	29	21	16	12	9	7	5	4
120	57	36	25	19	14	10	8	6	4	3

$40,000

# individuals \ # cells	500	750	1000	1250	1500	1750	2000	2250	2500	2750
40	308	195	138	103	80	69	57	48	43	38
48	251	157	109	80	60	53	43	39	34	30
56	206	130	92	68	53	44	36	31	27	24
64	178	111	77	57	43	36	30	26	22	19
72	150	94	66	49	38	31	25	21	18	16
80	133	83	58	43	32	27	22	18	15	13
88	114	72	51	38	29	23	19	16	13	11
96	104	65	45	33	25	20	16	13	11	9
104	90	56	40	30	23	18	14	11	9	7
112	83	52	36	27	20	16	12	9	7	6
120	72	45	32	24	18	14	11	8	6	5

$45,000

# individuals \ # cells	500	750	1000	1250	1500	1750	2000	2250	2500	2750
40	354	223	158	119	92	80	66	56	51	44
48	288	179	125	92	69	61	50	45	40	35
56	238	150	106	79	61	51	42	37	32	28
64	205	128	89	65	49	42	36	31	26	23
72	175	110	77	58	44	37	30	26	22	19
80	155	97	67	50	37	32	26	22	19	16
88	135	85	60	44	34	28	23	19	16	14
96	122	76	53	39	29	24	20	16	14	11
104	107	67	47	35	27	21	17	14	12	10
112	98	61	43	31	24	19	15	12	10	8
120	87	54	38	28	22	17	14	11	8	7

$50,000

# individuals \ # cells	500	750	1000	1250	1500	1750	2000	2250	2500	2750
40	399	252	178	134	104	90	75	63	58	50
48	325	202	141	103	78	69	56	51	46	40
56	271	170	120	90	69	59	48	42	37	32
64	233	145	101	74	56	48	41	36	31	27
72	200	126	88	66	50	42	34	30	26	23
80	178	111	77	57	43	37	30	26	22	19
88	155	97	68	51	39	32	27	22	19	16
96	140	88	61	45	34	28	23	19	16	14
104	124	78	55	41	31	25	21	17	14	12
112	114	71	50	37	28	22	18	15	12	10
120	102	64	45	33	25	20	16	13	11	9

Supplementary Figure 10: Coverage (thousands of reads per cell) across different budgets. Library preparation costs of $2,000 per reaction and greedy multiplexing (with at most 24,000 cells per reaction) are assumed. Colour scales correspond to the magnitude of the values in each cell of the heatmap.

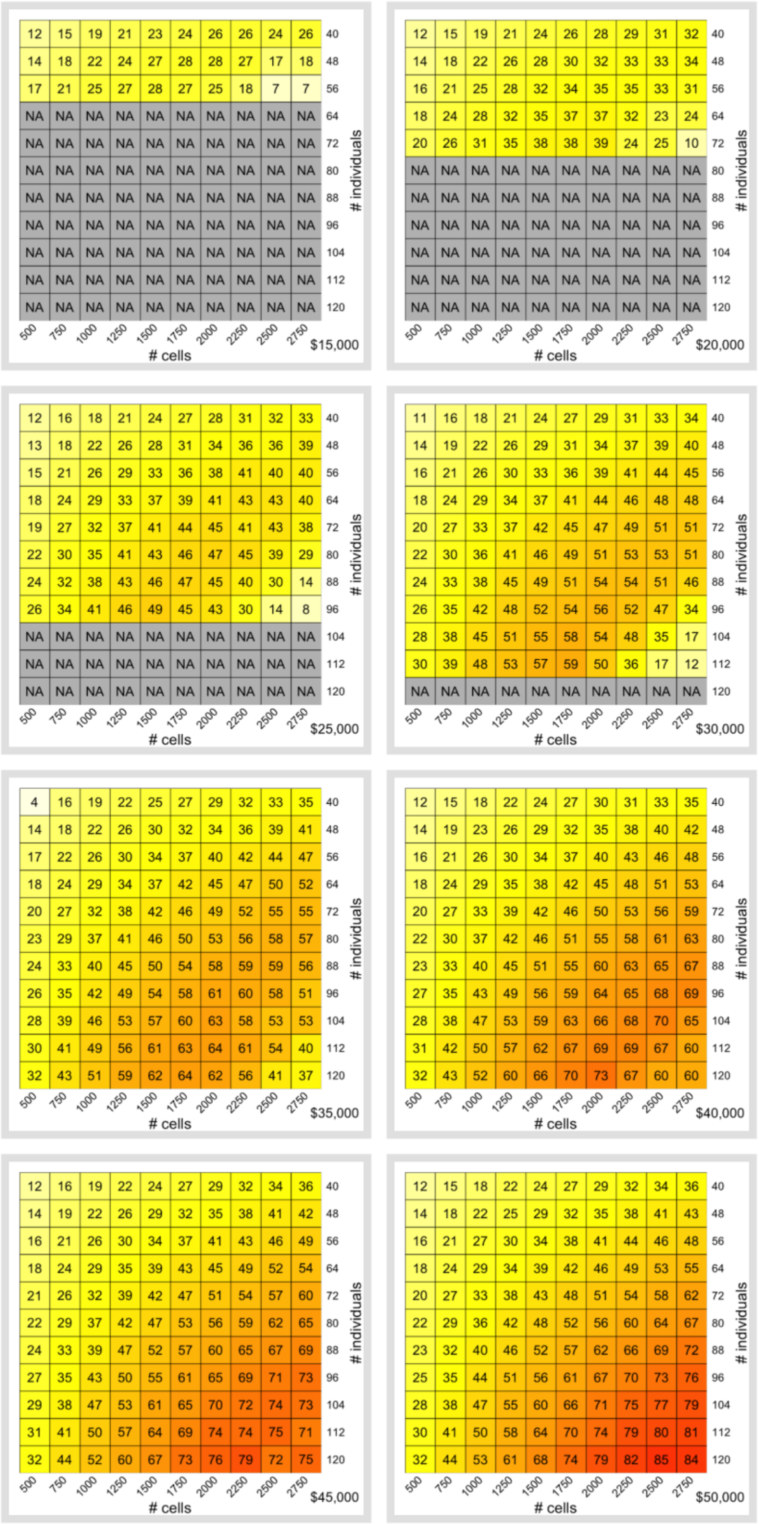

Supplementary Figure 11: Effective sample size for CD4 T cells across different budgets assuming library preparation costs of $2,000 per reaction and greedy multiplexing. Demultiplexing inaccuracy at low coverage is taken into account. Colour scales correspond to the magnitude of the values in each cell of the heatmap.

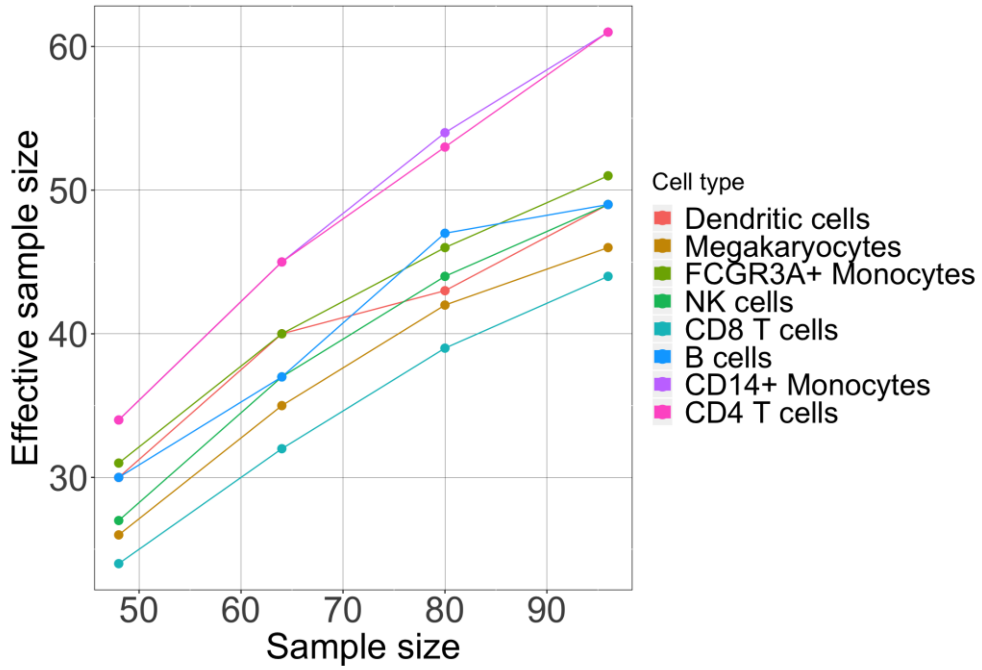

Supplementary Figure 12: Effective sample size for CD4 T cells as a function of sample size across different cell types at $35,000.

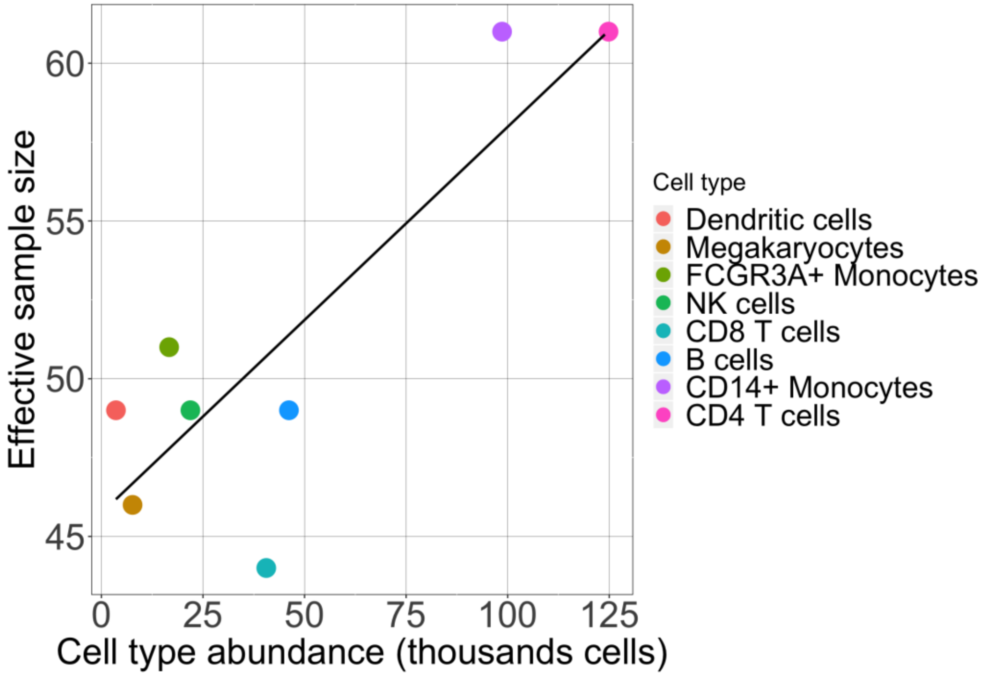

Supplementary Figure 13: R^2 between cell type abundances and the effective sample size is equal 0.72 (N = 96, B = $35,000).

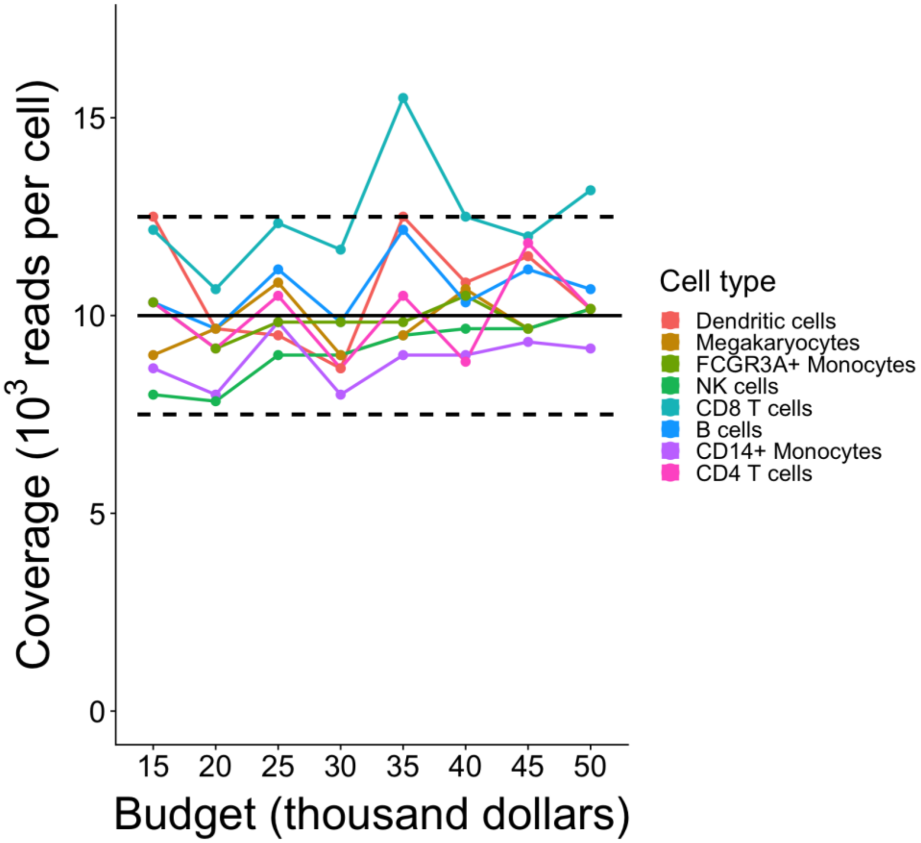

Supplementary Figure 14: Average coverage across top 3 most powerful ct-eQTL studies as a function of budget. Coverage corresponding to the experiments with the highest effective sample size (in the search space defined in the main text of the paper) is 10,000 $\pm$ 2,500 reads per cell.

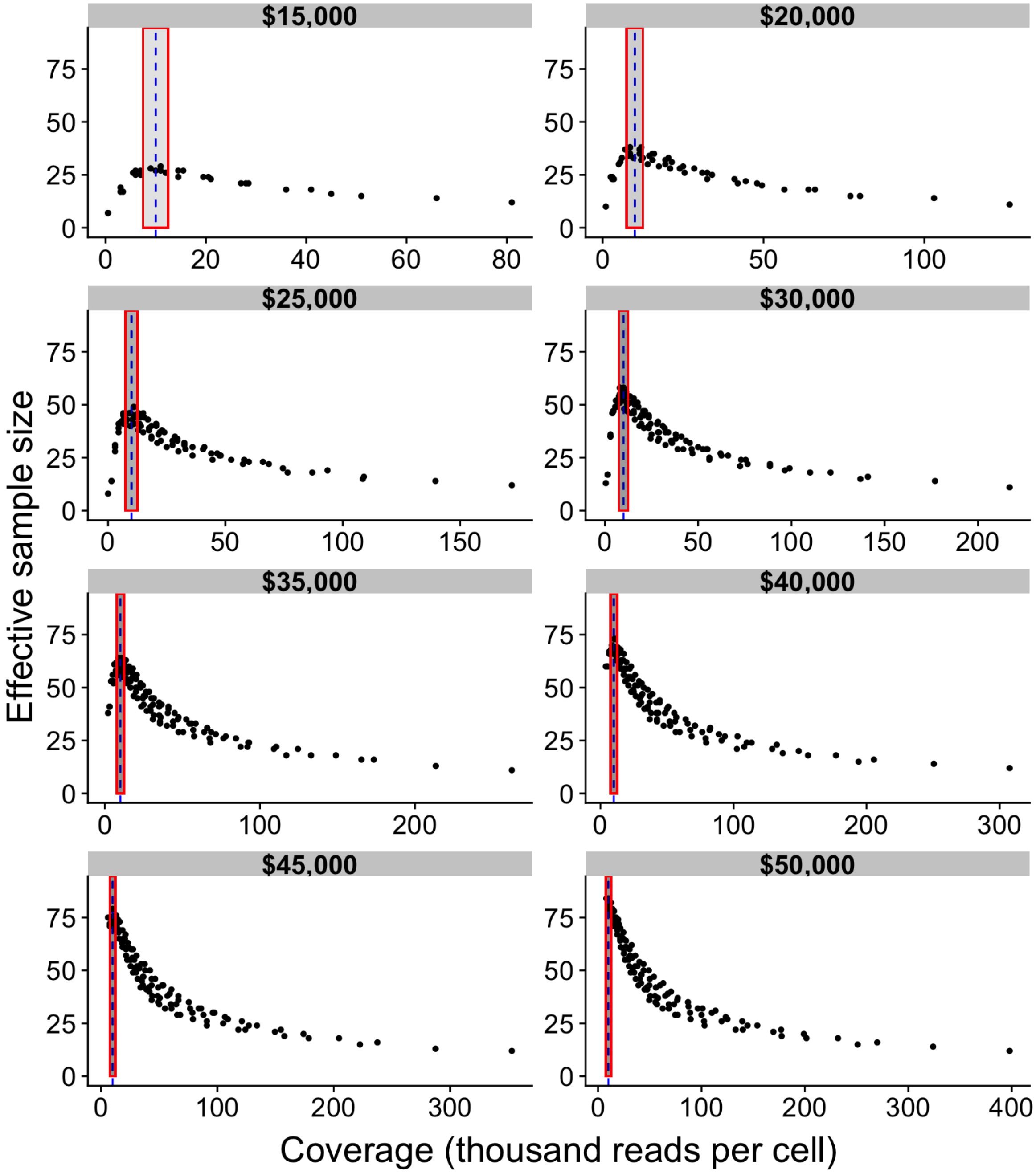

Supplementary Figure 15: Effective sample size as a function of read coverage (CD4 T cells). The optimal effective sample size is achieved at 10,000 $\pm$ 2,500 reads per cell. Each dot represents an experiment, the grey strip delimits the optimal coverage, i.e. the coverage at which the highest effective sample size is achieved.

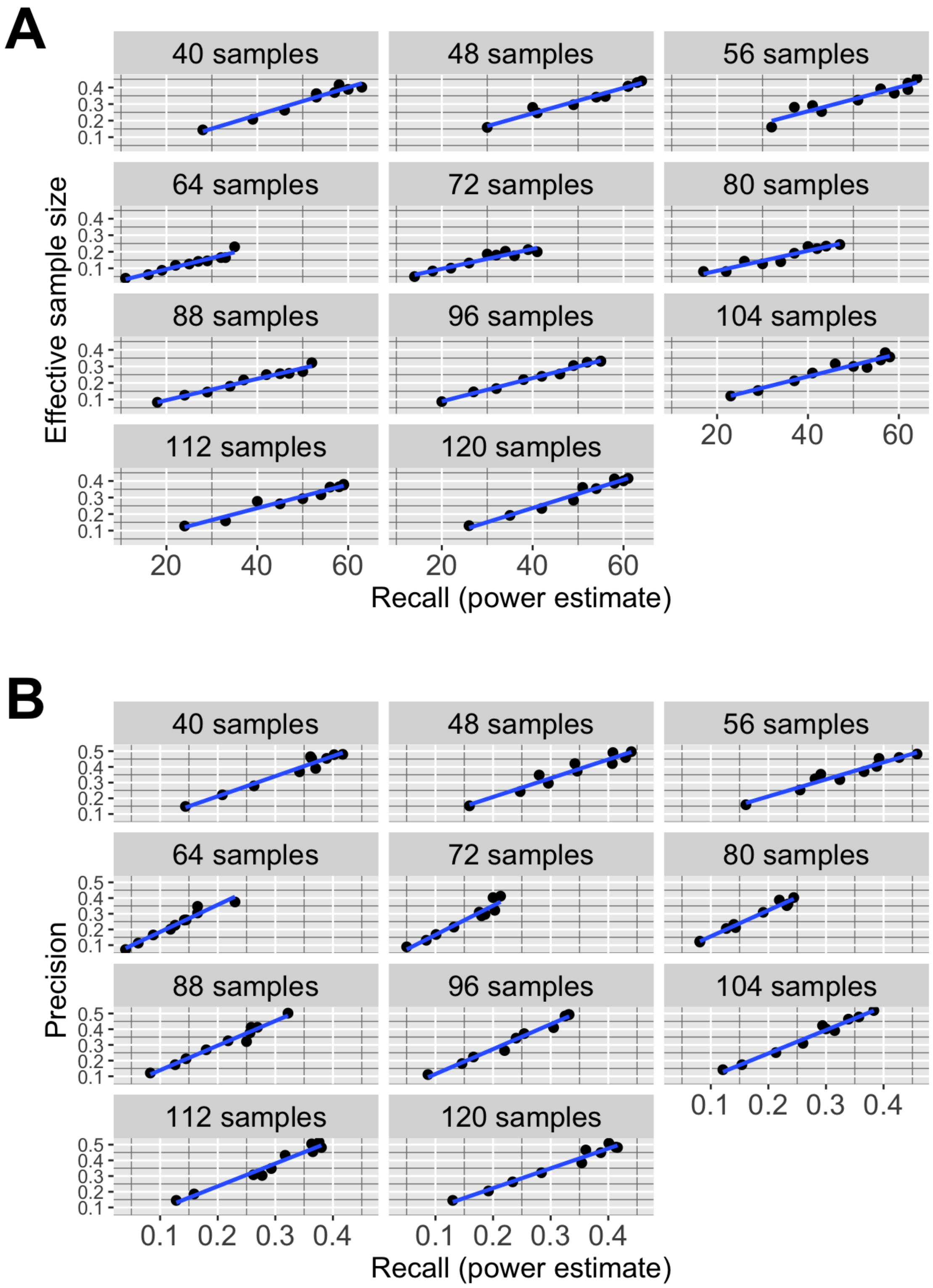

Supplementary Figure 16: Power estimates in ct-eQTL studies (CD4 T cells) stratified by sample size. A) Recall (power estimate) as a function of effective sample size. B) Precision-recall plot.

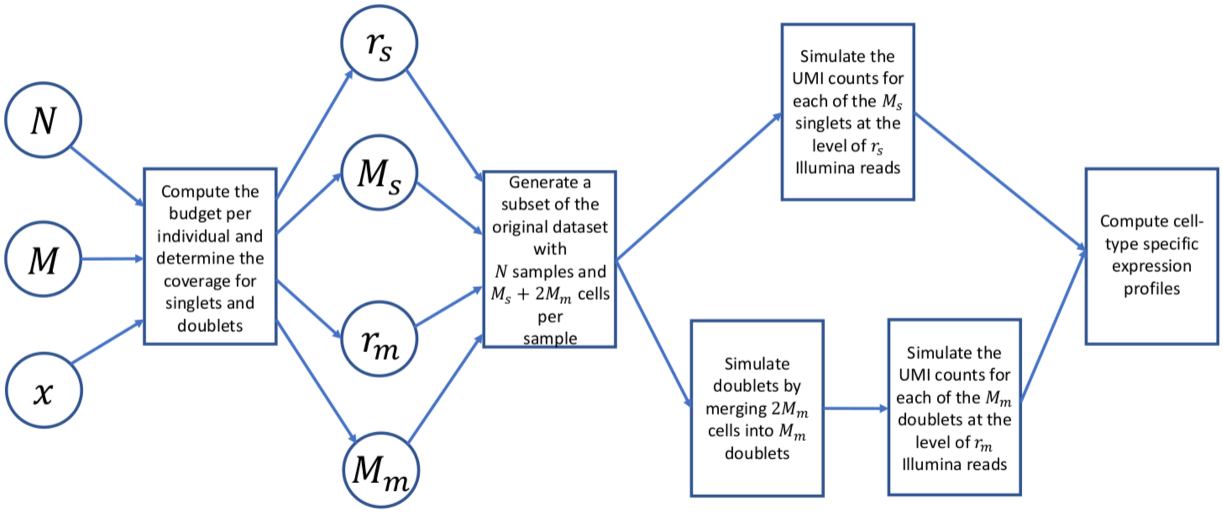

Supplementary Figure 17: The simulation workflow. The input to the simulation are the parameters N- the sample size, M- the number of cells per sample, x - multiplexing level. We first compute the budget per each individual. Then, by using the Satija lab single-cell cost calculator (https://satijalab.org/costpercell) we compute the number of singlets M_s with the coverage r_s and the number of multiplets M_m with the coverage r_m. We then randomly "merge" the expression profiles of $2M_m$cells pairwise to obtain M_m doublets. Finally, the UMI counts are simulated from each cell and the cell-type specific expression is computed for each cell type across all of the samples.

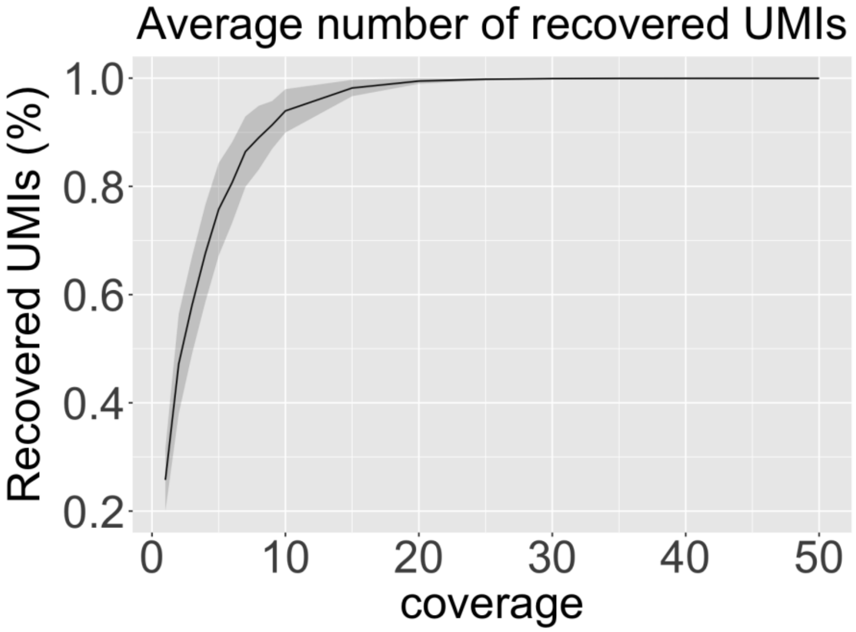

Supplementary Figure 18: Percent of total detected UMIs at different levels of coverage. At each level of coverage r, we randomly sampled a cell from the 10X dataset and simulated r read counts. The average number of recovered UMIs per cell (± 1 standard deviation) is computed over 1000 iterations.

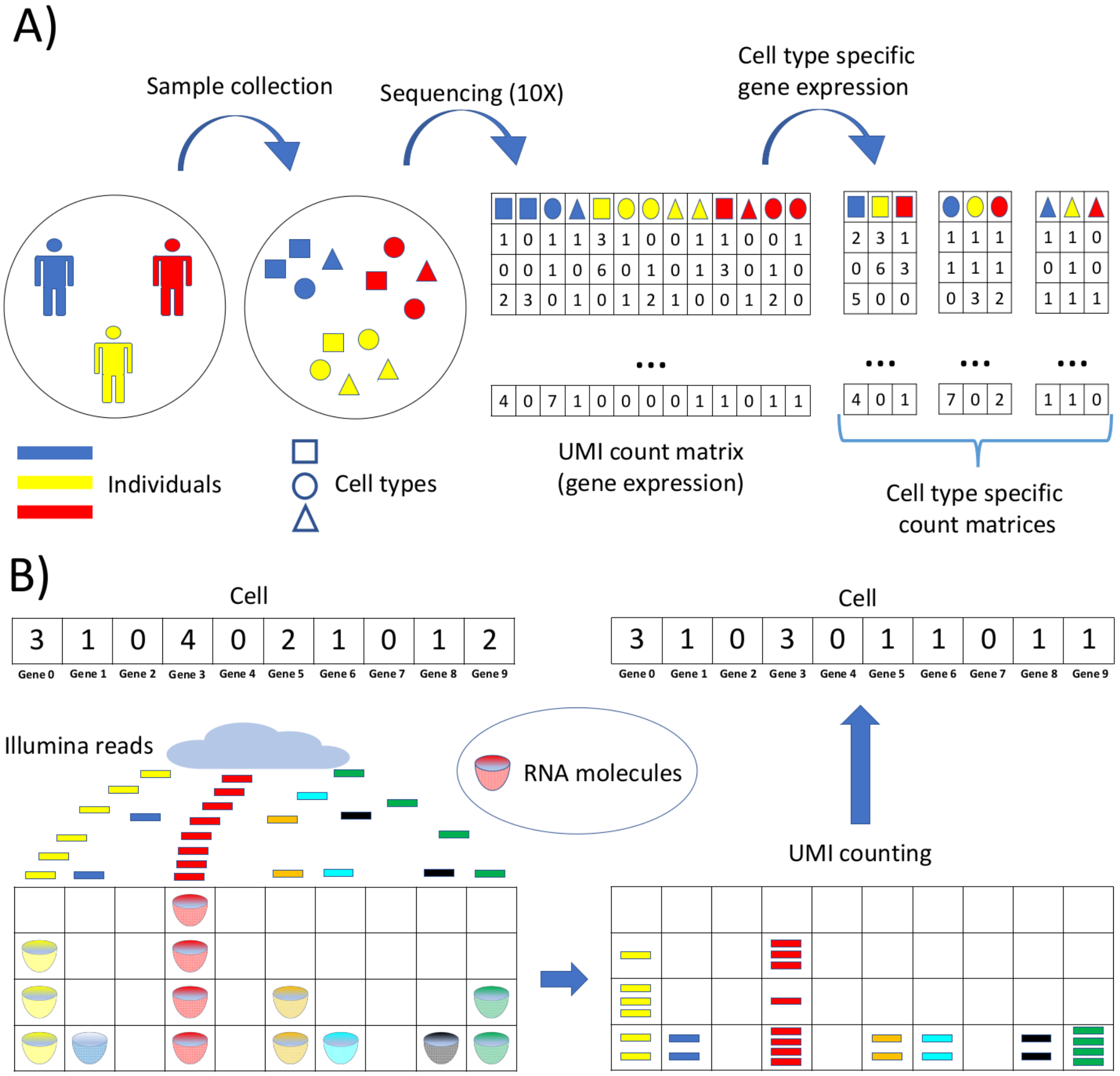

Supplementary Figure 19: Read count simulation for 10X. A) First, samples are collected, then sequenced by using 10X Genomics technology. Second, cell-type-specific gene expression is determined for each of the individuals. B) Each RNA molecule (or, equivalently, each UMI) regardless of the gene it was transcribed from is an urn, and each Illumina read is a ball which is randomly thrown into the urns. Given the high-coverage expression of a cell (on the left) as the number of RNA molecules in the high-coverage, the simulation of gene expression under a specified level of read coverage is performed as a random throwing of reads ("balls") into the corresponding RNA molecules ("urns"). After all the "balls" are thrown into the "urns", some urns remain empty (the gene received 0 reads, consequently, was not sequenced). In case a small number of reads is thrown into the "urns", a considerable number of so-called "drop-out" events (i.e., missing all UMIs from a gene) will occur.

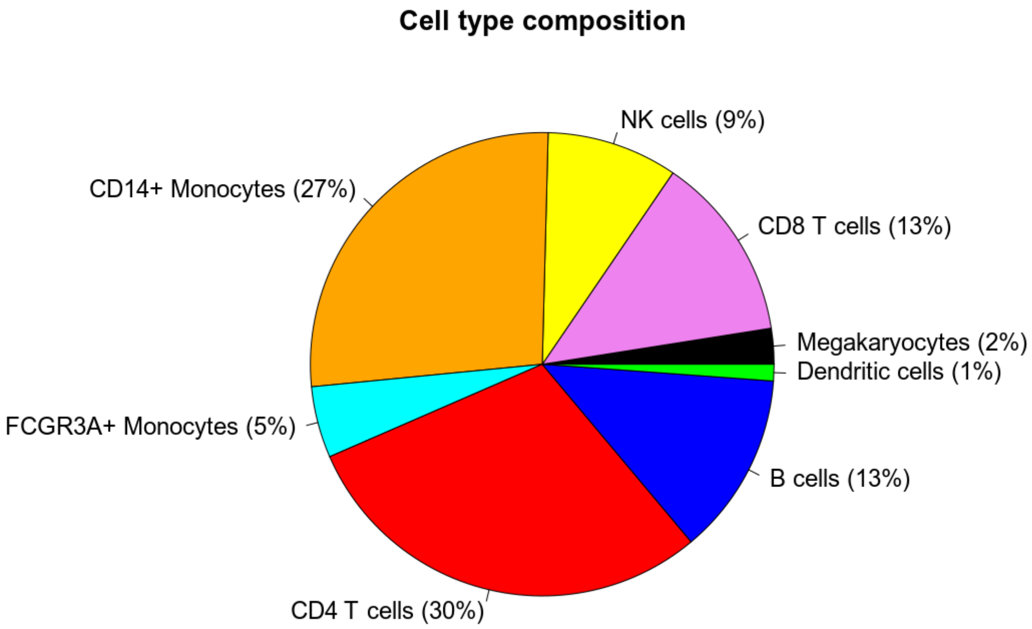

Supplementary Figure 20: Cell type composition of the 10X dataset.
