# Supplementary material for: Optimized design of single-cell RNA sequencing experiments for cell-type-specific eQTL analysis
Source: Nat Commun. 2020 Oct 30;11:5504. doi: 10.1038/s41467-020-19365-w (PMC7599215; doi:10.1038/s41467-020-19365-w)
Supplement: Supplementary file 2 — Peer Review File [file 41467_2020_19365_MOESM2_ESM.pdf]

Reviewers' comments:

Reviewer #1 (Remarks to the Author):

The authors present a guide for designing scRNA-sequencing studies for cell-type-specific eQTL analysis. Currently there exists little direction as to how to design these studies: few have been conducted and the expense remains high. Their work provides straightforward analyses that may help to guide the theory of designing such an experiment. However, the work here avoids any of the truly interesting aspects of single-cell analysis by framing the analysis as how does one get bulk estimates of purified cell types from a single-cell experiment. Major questions as to how this may impact variance-based QTL analyses or network-based/co-expression QTL analyses within single cells (i.e. where two genes are highly coupled in their regulatory effects explored in PMID: 29610479) need to be covered. Furthermore, there is no explicit modeling of cell-type prevalence as a function of the number of different cell-types in the pool or an assigned number of clusters. What would be the impact if one was to do cell-type specific eQTL analysis on 3 clusters/cell-types versus 10 clusters/cell-types present in a sample? Overall, the manuscript is well written but overly simplified reducing its impact and not having sufficient enough advance in its present form.
I have the following minor comments:
1) In the abstract the authors state that cell composition and state are “impossible” to measure in bulk profiling. This is too strong an assertion given the variety of computational cell-type deconvolution methods that are available.
2) The references 1-3 for single cell RNA-seq’s increased used over bulk do not fit with where they are referenced.
3) References 8-10 for eQTL mapping seem arbitrarily selected. Reference 8 and 9 are from GTEx with reference 9 having an incorrect or repeated author line. Reference 10 is regarding TWAS and not eQTL. There is a recent review by Leonid Kruglyak that could be used here instead in combination with the GTEx reference 9.
4) The equation for the budget model has terms subsequently described in a random order reducing the clarity. There is also a correction of 10e-6 when the r term could be read depth in millions of reads for simplicity.
5) Please refer to cost consistently. I.e. as p the cost of sequencing per 1 million reads. Later it appears as numbers such as $5000 for sequencing and this reduce interpretability with respect to the equation. When changing this please also include a calculation of how p was determined.
6) In practice, one may be limited to a particular number of reads. i.e. what can be achieved with a S2 flow-cell or a NextSeq flow cell. The equations do not appear to account for design as a function of this fixed cost property.
7) The plots in Fig 2 for Neff = 45 seem to have selected a sample size that is unusual for typical eQTL studies. Many of these studies are at least 60 individuals if not upwards of 100 individuals now. Is the minimum sample size 45 for a single-cell RNA-seq eQTL study?
8) Figure 3 is difficult to interpret. Please change colors to reflect increasing sample sizes and symbols to highlight differences in coverage.
9) Reference 22 is not appropriately listed and refers to a PubMed entry.

Reviewer #2 (Remarks to the Author):

Mandric et al. introduce a framework for determining the optimal experimental design for cell-type-specific eQTL studies utilizing scRNA-seq. Their framework provides some insights that will help guide researcher thinking about these types of studies, with the key conclusion being that in general power is maximized by profiling more individuals and more cells with fewer reads per cell, rather than fewer cells with more reads per cell. The authors show this through some convincing downsampling analyses using a high-quality Smart-seq2 dataset from pancreatic cells, and using a 10x Genomics dataset containing PBMCs from 120 individuals. Overall the manuscript is clear and well presented, and will be

a valuable resource for the community.

I am interested in further discussion of how power changes as a function of cell type frequency. For rare cell types, I would imagine the gene expression quantification accuracy would be lower (as there are fewer cells observed for each individual), and so the resulting Neff for the rare population would be lower than that of other cell types. This is mentioned briefly in discussion of the rare dendritic cells, but the implications of this could be explored/discussed in more detail in the manuscript.

Since genetic multiplexing of samples is suggested in order to reduce the cost of library production per sample and eliminate doublets, some exploration of the impact of low read coverage on the performance of genetic demultiplexing algorithms such as demuxlet and souporcell is needed. If low coverage prevents accurate demultiplexing of cells, this would alter the optimal experimental design.

To make this resource more accessible to the community, it would be great if the authors could provide their budget model as a web app (similar to the howmanycells or costpercell app from the Satija lab) where users can input different budgets or find the budget for a desired effective sample size. I think this would be very useful but should not be considered a prerequisite for publication.

Other comments:
* Since sample specific barcodes are mentioned in the introduction in the context of scRNA-seq sample multiplexing, the authors should consider citing the cell hashing work or MULTI-seq work in the introduction
* Bm should be defined in the main text where it is introduced
* Figure 3: I suggest thinking about different ways to visualize this, perhaps using faceting rather than different shapes for the points to make the difference due to sample size more visually clear
* It’s unclear where the 120-donor PBMC dataset originates from. No citation is given, was it generated in this study? If so, no methods are given.
* Some of the supplemental figure captions can be better described. For example, the caption for figure S12 seems to be incorrect, as the budget in each panel varies. It should also be explained more clearly what “coverage” refers to here.
* Greedy multiplexing should be defined/explained
* It’s unclear why the 2750 cell PBMC 10x dataset was chosen as a reference for label transfer. There are much better PBMC datasets which would likely result in higher label transfer accuracy.
* The correct citation for Seurat label transfer is Stuart et al. 2019, Cell
* More details should be given in the methods. What parameters were used for computational analyses such as Seurat label transfer, and what software versions were used?
* I suggest using a different word for “recommended design”, as this is not the design being recommended in the paper. “Naive” or “standard” for example might convey the message better.

Reviewer #3 (Remarks to the Author):

Overall comments

Mandric et al. present a series of simulations to demonstrate the effect of experimental design choices for single-cell RNA sequencing (number of samples, number of cells per sample, read depth) on statistical power in the context of cell-type-specific expression quantitative trait loci (eQTL) mapping. The aim is to provide practical guidelines for readers regarding experimental design. However, in its present form, the study has several major limitations, which need to be addressed to meet this aim.

Major comments

1) Figure 1 displays results showing that cell-type-specific gene expression is accurately recovered by

low-coverage sequencing. However, the evidence provided in this section is not strong. In particular, the results in Figure 1 rely on a single external dataset (Smart-seq2, 2209 pancreatic cells from 10 individuals), and have been summarized across genes. To strengthen the evidence, it would be useful to investigate additional datasets (e.g. from other protocols, as well as additional cell types), and to provide some illustrative results for individual genes (e.g. scatterplots demonstrating that the use of Pearson R^2 is appropriate, i.e. linear relationships).

Also in this section, a threshold of 70% average R^2 is used to establish that downsampled data accurately reflect the full data. This threshold seems arbitrary – could the authors provide some justification for this threshold?

Figure 1B uses log-transformed transcripts per million (TPM) for mean expression – could the authors justify this choice?

2) Insufficient technical detail is provided to justify and explain the "effective sample size" concept (N_eff; Pearson R^2 multiplied by sample size), which is used as a key evaluation metric throughout the study. (Two references are provided – refs. 25 and 26 – but these also do not provide much detail.) Since most of the results rely on this metric, it needs to be clearly explained and motivated, including for readers who are not familiar with the concept.

3) The authors state that a key aim is to provide practical guidelines for designing ct-eQTL studies, and several strong statements are made (e.g. regarding optimal statistical power). However, these results mainly refer to the examples in Figures 2 and 3, which present specific guidance for these datasets. It is not clear how readers can translate this guidance to new datasets.

For example, Figure 2 shows optimal design parameters for the B cell dataset with effective sample size 45. How would readers perform similar design calculations for a new dataset? Would the specific recommendations change for different protocols or different numbers of cells? (These results also rely again on the effective sample size N_eff – how would this be calculated for a new dataset?) Could the authors also provide more details on the definition of optimality, especially in the context of new datasets?

4) Most of the results rely on the use of known reference cell types. However, for many datasets, this information is not available. The authors provide some results on uncertainty due to cell type identification and briefly mention this limitation in the discussion. However, this is a major limitation, which will affect many readers – could the authors expand on this?

5) Have the authors considered the use of imputation techniques to further reduce the number of required cells per sample?

6) Code and data to reproduce the results have not been provided. Code and data repositories (e.g. GitHub, Zenodo, etc.) should be provided both for reproducibility purposes and to allow readers to adapt the calculations for new datasets.

Minor comments

When specifying the number of cells per sample, does this refer to the final number of measured cells, or the number of input cells?

To provide practical guidance for readers, the authors could also consider creating an online calculator, e.g. using R/Shiny.

Figure 1: which cell type is "cell type 1"?

Figure 2: please label panels A and B

Figure 2B: please provide axis labels

Figure 2A: why does the "recommended" design use 33,000 reads per cell, instead of 50,000 as mentioned in the text?

Results, section 2: justification (or a reference) should be provided for the statement "since according to demuxlet, 99% of the sample identities can be correctly recovered at this level of multiplexing".

Results, section 2: why is the per-reaction capacity limited to 24,000 cells? (e.g. provide reference)

Supplementary figures are not referred to in sequence in the text.

Response to reviewers for *Mandric et al.*

We thank the reviewers for insightful comments that have allowed us to greatly enhance the quality of our manuscript. You will find that the revised manuscript answers all comments and concerns raised by reviewers and as such is significantly improved. In particular we have:

- Estimated demultiplexing inaccuracy at low-coverage and re-ran the experiments with the updated pipeline which takes into account this inaccuracy
- Showed that the optimal coverage for cell-type-specific eQTL studies is 10,000 reads per cell (no matter the budget and cell type)
- Analyzed the performance of variance cell-type-specific eQTL analysis in the framework of the existing work and showed that it is also optimal to use 10,000 reads per cell
- Included the analysis of a 10X Genomics single-cell dataset based on the Census of Immune Cells to show that at low-coverage one can accurately estimate the cell-type-specific gene expression (given enough cells for each individual)
- Created a web app calculator for computing the effective sample size and finding the optimal experimental design.

For your convenience, we provide a point-by-point response to all comments raised by reviewers in blue font below.

Bogdan Pasaniuc (on behalf of all co-authors).

——————-

Reviewer #1

The authors present a guide for designing scRNA-sequencing studies for cell-type-specific eQTL analysis. Currently there exists little direction as to how to design these studies: few have been conducted and the expense remains high. Their work provides straightforward analyses that may help to guide the theory of designing such an experiment. However, the work here avoids any of the truly interesting aspects of single-cell analysis by framing the analysis as how does one get bulk estimates of purified cell types from a single-cell experiment.

1) Major questions as to how this may impact variance-based QTL analyses or network-based/co-expression QTL analyses within single cells (i.e. where two genes are highly coupled in their regulatory effects explored in PMID: 29610479) need to be covered.

We thank the reviewer for this comment. Based on the suggestion of the reviewer, we now explored how low-coverage scRNA-sequencing may impact variance-based QTL analyses, and we found that the results are similar to the case of mean-based QTL analysis. We also added a figure (Figure 5B), summarizing these results. We have now added the following text (Page 12 in the Results section):

"Additionally, we explored cell-type specific variance eQTL analysis as opposed to cell-type specific mean eQTL. We restricted our analysis to CD4 T cells (the most abundant cell type) at $B = \$35,000$. For this level of budget, similar to the above analysis of mean ct-eQTL, we performed variance ct-eQTL analyses for each experimental design (N, M, r), where N ranged from 40 to 120 with step 8 and M ranged from 500 to 2,750 with step 250. We also ran the

variance ct-eQTL analysis on the original dataset to obtain the "ground truth" set of variance ct-eQTLs. As in the case of mean eQTL, low-coverage experiments yield higher power for variance eQTL analysis (Figure 5B). "

2) Furthermore, there is no explicit modeling of cell-type prevalence as a function of the number of different cell-types in the pool or an assigned number of clusters. What would be the impact if one was to do cell-type specific eQTL analysis on 3 clusters/cell-types versus 10 clusters/cell-types present in a sample?

We thank the reviewer for this insightful comment. Based on the reviewer's suggestion, we have added another experiment, in which we performed the entire analysis taking the prevalence of the cell-types into account. Specifically, for different values of cell-type prevalence we searched for the best study designs, and we report the tradeoff between the cost and the prevalence in the Results section. In particular, we added a figure (Figure 4, main text) that summarizes these results, and added the following text to the Results (Page 10, main text):

"To study how effective sample size depends on cell-type prevalence, we simulated datasets with different levels of CD4 T cells prevalence ranging from 5 to 30% (Figure 4). As expected, for one particular cell type, effective sample size is a monotonic function of prevalence (given all other parameters are fixed). Nevertheless, the highest effective sample size is achieved at coverages around 10,000 reads per cell irrespective of cell-type prevalence."

3) Overall, the manuscript is well written but overly simplified reducing its impact and not having sufficient enough advance in its present form.

We thank the reviewer for this comment. We now revised our paper in order to make sure we increase the impact of the manuscript and to further advance its main ideas. Indeed, in the new revision of our paper we:

- Analyzed cell-type-specific variance eQTLs and showed that the results are similar to cell-type-specific mean eQTLs;
- Included modeling of demultiplexing inaccuracy at low read coverages;
- Provided the analysis of how statistical power changes as a function of cell-type prevalence.

Minor comments:

1) In the abstract the authors state that cell composition and state are "impossible" to measure in bulk profiling. This is too strong an assertion given the variety of computational cell-type deconvolution methods that are available.

We agree with the reviewer, and we now modified the text of the manuscript as follows (Page 2, Abstract):

"Single-cell RNA-sequencing (scRNA-Seq) is a compelling approach to directly and simultaneously measure cellular composition and state, which can otherwise only be estimated by applying deconvolution methods to bulk RNA-Seq estimates."

2) The references 1-3 for single cell RNA-seq's increased used over bulk do not fit with where they are referenced.

We thank the reviewer for this comment. We agree that the references 1-3 did not fit with where they were referenced. We updated the references as follows:

"Massively parallel single-cell RNA sequencing (scRNA-Seq) has been increasingly used over the past few years as a powerful alternative to bulk RNA-Seq. Key advantages of scRNA-Seq over bulk methods are the ability to reveal complex and rare cell populations[1–5], uncover regulatory relationships between genes[6–8], and track the trajectories of distinct cell lineages in development[9,10]."

References 1-5 emphasize the ability of scRNA-Seq to reveal cell types:

- Guerrero-Juarez et al., 2019
- Karamitros et al., 2018
- Hernandez et al., 2018
- Grun et al., 2015
- Villani et al., 2017

References 6-8 help supporting the statement that scRNA-Seq uncovers regulatory relationships between genes:

- Bendall et al., 2014
- Shema et al., 2019
- Kakaradov et al., 2017

Finally, references 9 and 10 bring the evidence for the fact that scRNA-Seq is used to track the development of distinct cell lineages:

- Rizvi et al., 2017
- Pollen et al., 2014

3) References 8-10 for eQTL mapping seem arbitrarily selected. Reference 8 and 9 are from GTEx with reference 9 having an incorrect or repeated author line. Reference 10 is regarding TWAS and not eQTL. There is a recent review by Leonid Kruglyak that could be used here instead in combination with the GTEx reference 9.

We thank the reviewer for this comment. We updated the references. In the main text (Page 3, Introduction), we included the following two references:

- (GTEX consortium, 2017)
- (Albert et al., 2018)

These are now references 14 and 15:

"Expression quantitative trait loci (eQTL) mapping is a widely-used tool in functional genomics used to identify mechanisms underlying the genotype-to-disease connection[14,15]."

4) The equation for the budget model has terms subsequently described in a random order reducing the clarity. There is also a correction of 10e-6 when the r term could be read depth in millions of reads for simplicity.

We thank the reviewer for this comment. Indeed, in the initial version of the paper, the equation for the budget model was not very clear. Therefore, we modified the equation by grouping 10^{-6} with p (Pages 7 and 14 of the main text):

$$B = B^m + \frac{N \cdot L}{x} + (10^{-6} \cdot p) \cdot N \cdot M \cdot r$$

5) Please refer to cost consistently. I.e. as p the cost of sequencing per 1 million reads. Later it appears as numbers such as $5000 for sequencing and this reduces interpretability with respect to the equation. When changing this please also include a calculation of how p was determined.

We thank the reviewer for this comment. In the main text of the paper, $5,000 that are allocated for sequencing refers to the <u>overall</u> costs of sequencing and not to the cost per 1 million reads (which is the value of the parameter p). In order to improve the readability of the manuscript, we clarify that $5,000 is the cost of sequencing 1 billion reads at $p = \$5$, (cost per million reads). We modified the text as follows (Page 7, main text):

"In all our experiments, the search space is defined by N ranging from 40 to 120 individuals in steps of 8 and M ranging from 500 to 2,750 cells per individual in steps of 250. Specifically, for 120 individuals, if each pool contains eight individuals, resulting in 15 pools, and the cost of library preparation per reaction is $L = \$2{,}000$, one needs to spend $30,000 for library preparation. Additionally, when the average cost of Illumina sequencing per 1 million reads is set to $p = \$5$, one needs to spend $5,000 for sequencing 1 billion reads. With this amount of sequencing, when $N = 120$ and $M = 2750$, each cell is sequenced with $r \approx 3000$ reads which is considered an extremely low coverage. Therefore, we fix the budget at $B = \$35{,}000$."

6) In practice, one may be limited to a particular number of reads. i.e. what can be achieved with a S2 flow-cell or a NextSeq flow cell. The equations do not appear to account for design as a function of this fixed cost property.

We thank the reviewer for this valuable comment. We do agree that for a more precise modeling of sequencing costs one has to include such parameters as the number of reads that can be achieved with a particular flow-cell (like S2 or NextSeq). However, a fine-grained modeling of sequencing costs would inevitably lead to dissipating the main message of our paper (single-cell experimental designs). In our model, parameter p stands for the average sequencing cost per 1 million reads which should account for the throughput of flow-cells and/or other particular characteristics of sequencing. In order to make this clear, we modified the text of the paper (Page 7, main text):

"p is the average cost of Illumina sequencing per 1 million reads (in dollars), L is the library preparation cost per reaction, and B^m is the budget wasted on sequencing of identifiable multiplets. B^m is an increasing nonlinear function of N, M, and x (for more details see Methods). Note that in the budget model we do not consider the details of the sequencing process (e.g., fixed flow-cell capacity, for example) but let p account for that."

However, we do recognize that this is a caveat of our paper. We explain this in the Discussion section of the main text (Page 13, main text):

"Second, the budget model used in our paper assumes that sequencing cost per one million reads is a fixed parameter p. However, this assumption hides the underlying details of sequencing such as, for example, flow-cells (S2, NextSeq, etc) used in the experiments. Flow-cells have different output capacities, and therefore, the number of flow-cells used in the experiment may vary, affecting the average costs per one million reads. For a more fine-grained budget modelling of scRNA-Seq, one has to account for such details and, therefore, use a more sophisticated (and more realistic) budget model."

7) The plots in Fig 2 for Neff = 45 seem to have selected a sample size that is unusual for typical eQTL studies. Many of these studies are at least 60 individuals if not upwards of 100 individuals now. Is the minimum sample size 45 for a single-cell RNA-seq eQTL study?

We thank the reviewer for this comment. Sample sizes in Figure 2 (for Neff = 45) in the previous version of the manuscript were not usual for a typical eQTL studies using bulk RNA-Seq data. However, for ct-eQTL studies using scRNA-Seq data, these sample sizes are large due to prohibitively high costs. The 10X dataset that we used (accession ID: GSE137029) was the largest in terms of the sample size at the time of writing the manuscript. However, taking into consideration the major comment 2 of the Reviewer #2 (about modeling demultiplexing

inaccuracy at low read coverage) we modified the simulation pipeline and re-ran the experiments. Also, we considered CD4 T cells instead of B cells (to be consistent with the other experiments throughout the manuscript). Based on the suggestion of the reviewer to include experiments with sample size at least 60 individuals, we updated Figure 2 to showcase experiments with 56, 64, 72, 80, and 88 sample sizes. Finally, Figure 2 has changed its number and it is now Figure 3 in the main text.

8) Figure 3 is difficult to interpret. Please change colors to reflect increasing sample sizes and symbols to highlight differences in coverage.

We thank the reviewer for the suggestion to improve the quality of Figure 3. We updated the figure according to the suggestions (this figure is Figure 5 now).

9) Reference 22 is not appropriately listed and refers to a PubMed entry.

We thank the reviewer for pointing out the wrong format of the reference. We updated the reference to (Kang et al., 2018).

Reviewer #2

Mandric et al. introduce a framework for determining the optimal experimental design for cell-type-specific eQTL studies utilizing scRNA-seq. Their framework provides some insights that will help guide researcher thinking about these types of studies, with the key conclusion being that in general power is maximized by profiling more individuals and more cells with fewer reads per cell, rather than fewer cells with more reads per cell. The authors show this through some convincing downsampling analyses using a high-quality Smart-seq2 dataset from pancreatic cells, and using a 10x Genomics dataset containing PBMCs from 120 individuals. Overall the manuscript is clear and well presented, and will be a valuable resource for the community.

1) I am interested in further discussion of how power changes as a function of cell type frequency. For rare cell types, I would imagine the gene expression quantification accuracy would be lower (as there are fewer cells observed for each individual), and so the resulting Neff for the rare population would be lower than that of other cell types. This is mentioned briefly in discussion of the rare dendritic cells, but the implications of this could be explored/discussed in more detail in the manuscript.

We thank the reviewer for this very important comment. We analyzed the dependence of power on cell type frequency. We came to the conclusion that the average effective sample size (across all the genes) is not a monotonic function of cell type frequency. This can be explained by different patterns in cell-type-specific expression and different architecture of cell-type-specific eQTL signals. However, when we fit a linear regression line, we observe that R^2 between the

cell counts and the effective sample size is relatively high (0.67). We modified the text of the manuscript as follows (Page 10, main text):

"We next analyzed the impact of cell type frequency on the power in a ct-eQTL study. In general, ct-eQTL studies in a more frequent cell type (for example, CD14+ Monocytes and CD4 T cells) have higher power. However, this dependency is not monotonic and linear (Figure S10, Supplementary). Nevertheless, R^2 between the cell counts and the effective sample size is high (for example, when B = \$35,000, N = 96 individuals, M = 2000 cells per individual, and r = 12,500 reads per cell, R^2 = 0.67 (Figure S11, Supplementary)). Despite the fact that the effective sample size is different for different cell types across different budgets, the optimal coverage (i.e. coverage, at which we observe the maximum effective sample size across the explored experimental designs) is 10,000 $\pm$ 2,500 reads per cell (Figure S12, Supplementary)."

2) Since genetic multiplexing of samples is suggested in order to reduce the cost of library production per sample and eliminate doublets, some exploration of the impact of low read coverage on the performance of genetic demultiplexing algorithms such as demuxlet and souporcell is needed. If low coverage prevents accurate demultiplexing of cells, this would alter the optimal experimental design.

We thank the reviewer for this very important comment. Based on the comment, we performed a new analysis with the goal of estimating the impact of low-coverage sequencing on demultiplexing. In more details, we used raw data (BAM files) for one 10X reaction (16 individuals) in order to estimate the quality of demultiplexing provided by the tool demuxlet at different levels of coverage. We downsampled the BAM files (ignoring multi-mapped reads) to 2, 4, 6, …, 30 thousand reads per cell (initial coverage was 30,000 reads per cell), ran demuxlet, and computed the percentage of cells which were assigned to the correct individual. Our analysis suggested that indeed, the performance of demultiplexing degrades with decreasing read coverage. This leads to a very important conclusion that one can increase the number of individuals and the number of cells per individual only up to a level (within a fixed budget) since an extremely low coverage would prevent accurate demultiplexing and would therefore negatively impact the accuracy of cell-type-specific gene expression thus reducing the power to call eQTLs. We created a new figure (Figure 2) which displays the dependence of demultiplexing accuracy on read coverage. Further, we modified our simulation pipeline by adding a preprocessing step when the sample labels of a subset of cells are randomly permuted (the size of the subset of cells depends on the read coverage according to the line fitted in Figure 2). Following the conclusions of the above described analysis, we also re-ran the whole grid of experiments (110 experimental designs with both cell-type identification and demultiplexing inaccuracies taken into account). We also modified the text of the paper as follows (Page 10, main text):

"To estimate demultiplexing accuracy at low coverage, we considered a subset of the 10X Genomics dataset (one reaction, 16 individuals). We then downsampled this dataset at different levels of coverage and ran demuxlet in order to compute $d = d(r)$ - the proportion of cells which

are assigned to the correct sample (Figure 2). Demultiplexing accuracy is high (~90%) at coverages $\geq 10{,}000$ reads per cell. When coverage is below that value, demultiplexing accuracy declines rapidly. To account for demultiplexing inaccuracy at coverage r, before analyzing the experimental design (N, M, r), we first randomly permute sample labels for $(1 - d(r))\,\%$ of cells."

3) To make this resource more accessible to the community, it would be great if the authors could provide their budget model as a web app (similar to the howmanycells or costpercell app from the Satija lab) where users can input different budgets or find the budget for a desired effective sample size. I think this would be very useful but should not be considered a prerequisite for publication.

We thank the reviewer for the idea of a web app. We have created an online calculator similar to the costpercell app from the Satija lab. Our app accepts two parameters: cell type and budget. The output of the calculator is the expected effective sample size and the list of possible experimental designs which can lead to this level of effective sample size. We host our calculator at: https://mandricigor.github.io/ct-eqtl-design/.

Minor comments:

1) Since sample specific barcodes are mentioned in the introduction in the context of scRNA-seq sample multiplexing, the authors should consider citing the cell hashing work or MULTI-seq work in the introduction

We thank the reviewer for pointing out the correct references. We included the references to the cell hashing (Stoeckius et al., 2018) and MULTI-Seq (McGinis et al., 2019) works (Page 4, main text).

2) Bm should be defined in the main text where it is introduced

We thank the reviewer for the comment. We updated the text of the manuscript as follows (Page 7, main text):

"B^m is the budget wasted on sequencing of identifiable multiplets. B^m is an increasing nonlinear function of N, M, and x (for more details see Methods)".

3) Figure 3: I suggest thinking about different ways to visualize this, perhaps using faceting rather than different shapes for the points to make the difference due to sample size more visually clear

We agree with the reviewer that Figure 3 of the previous version of the manuscript did not emphasize the difference due to sample size. We also thank the reviewer for helpful suggestions on improvement of Figure 3. In the updated version of the manuscript, this figure is moved to the

Supplementary (Figure S13). We used faceting to make the difference due to sample size more visually clear.

4) It's unclear where the 120-donor PBMC dataset originates from. No citation is given, was it generated in this study? If so, no methods are given.

We thank the reviewer for the comment. We have included the accession ID for the dataset used in the manuscript. We modified the text as follows (Page 7, main text):

"In what follows, we analyzed a 10X Genomics dataset (accession ID: GSE137029) consisting of 120 individuals each having 2,750 cells (see Methods)."

5) Some of the supplemental figure captions can be better described. For example, the caption for figure S12 seems to be incorrect, as the budget in each panel varies. It should also be explained more clearly what "coverage" refers to here.

We thank the reviewer for the rigorous review. We updated supplemental figure captions to better describe the figures. For example, Figure S9 (which previously was Figure S12) has now an updated caption:

"Figure S9: Coverage (thousands of reads per cell) across different budgets assuming library preparation costs of $2,000 per reaction and greedy multiplexing (with at most 24000 cells per reaction)."

6) Greedy multiplexing should be defined/explained

We thank the reviewer for the comment. As compared to multiplexing with a fixed number of samples per pool, greedy multiplexing refers to the case when we "pack" as many individuals as possible (but not exceeding 16) in a pool such that the total number of cells in the pool does not exceed 24,000. For example, we have 40 individuals with 1000 cells per individual. In the case of greedy multiplexing, we will not use 5 pools of 8 samples each, but rather 3 pools (14, 13, and 13 samples correspondingly). We modified the text of the paper as follows (Page 9, main text):

"A natural approach is to multiplex more individuals when possible (i.e., when M is not high). We refer to this approach as greedy multiplexing. We limit the per reaction capacity to 24,000 cells[23] and allow x to take on the values up to 16."

7) It's unclear why the 2750 cell PBMC 10x dataset was chosen as a reference for label transfer. There are much better PBMC datasets which would likely result in higher label transfer accuracy.

We thank the reviewer for the comment. We agree that some alternative datasets with larger number of cells or better annotations can be used for label transfer. However, the 2700 PBMC

dataset is readily available on the 10X webpage, it is highly reliable, and it is very popular among researchers. Also, due to its small size, the runtime of our simulations is not exceedingly high.

8) The correct citation for Seurat label transfer is Stuart et al. 2019, Cell

We thank the reviewer for pointing out the correct reference. We updated the citation for Seurat label transfer to (Stuart et al., 2019).

9) More details should be given in the methods. What parameters were used for computational analyses such as Seurat label transfer, and what software versions were used?

We thank the reviewer for the comment. We used the label transfer feature of Seurat as it is described on the webpage of Satija lab (https://satijalab.org/seurat/v3.0/integration.html - Standard Workflow). We updated the text of the manuscript as follows (Page 16, main text):

"Cell types were determined by using the label transfer feature from Seurat (version 3.0) using the default parameters[37]. As the reference, the 2700 PBMC 10X dataset was used[38]."

10) I suggest using a different word for "recommended design", as this is not the design being recommended in the paper. "Naive" or "standard" for example might convey the message better.

We thank the reviewer for the suggestion. We agree that the phrase "recommended design" is confusing since it is not clear whether this is the recommendation of our paper or a standard practice. We changed the word "recommended" to "standard" (Figure 3A).

Reviewer #3

Mandric et al. present a series of simulations to demonstrate the effect of experimental design choices for single-cell RNA sequencing (number of samples, number of cells per sample, read depth) on statistical power in the context of cell-type-specific expression quantitative trait loci (eQTL) mapping. The aim is to provide practical guidelines for readers regarding experimental design. However, in its present form, the study has several major limitations, which need to be addressed to meet this aim.

Major comments:

1) Figure 1 displays results showing that cell-type-specific gene expression is accurately recovered by low-coverage sequencing. However, the evidence provided in this section is not strong. In particular, the results in Figure 1 rely on a single external dataset (Smart-seq2, 2209 pancreatic cells from 10 individuals), and have been summarized across genes. To strengthen the evidence, it would be useful to investigate additional datasets (e.g. from other protocols, as well as additional cell types), and to provide some illustrative results for individual genes (e.g. scatterplots demonstrating that the use of Pearson R^2 is appropriate, i.e. linear relationships).

We thank the reviewer for the suggestion. To strengthen the evidence that cell-type-specific gene expression can be accurately inferred at low read coverage, we analyzed an additional 10X Genomics dataset which is a subset of the Census of Immune Cells (which is a part of Human Cell Atlas). The cells from this dataset come from bone marrow tissue. On this dataset, we perform a similar analysis with the analysis described in the Results section of the main text (Smart-Seq2 dataset). The analysis is described in detail in Supplementary Note 2. It reveals that with decreasing coverage, the R^2 between the inferred and ground truth gene expression is decreasing as well. However, even, for example, at 4,000 reads per cell, the average R^2 across 1341 genes is approximately 0.7 (Figure S22A). We added the following text to the main paper (Page 6, main text):

"To strengthen the evidence that cell-type-specific gene expression can be accurately inferred at low read coverage, we also analyzed a 10X dataset. Specifically, we downloaded Census of Immune Cells[12] dataset and considered a subset of it corresponding to bone marrow cells (we considered only lane 1, see Methods) consisting of 21,485 cells belonging to 8 donors. The initial read coverage of the dataset is approximately 20,000 reads per cell. We downsampled the reads at different levels of coverage (4,000 to 20,000 reads per cell with step 2,000) and computed cell-type-specific gene expression matrices. We restricted our analysis to the known 1341 marker genes encountered in bone marrow cell types[35]. We computed Pearson R^2 between the low-coverage estimates and the ground truth gene expression (i.e., the one computed at full coverage of 20,000 reads per cell). We observed that for example, at 4,000 reads per cell, the average R^2 in erythroblast cells is approximately 70% (Figure 1C; see some example genes in Figure S3, Supplementary). The average R^2 is high across all the genes (lowly- and highly-expressed, see Figure 1D). Most genes are expressed in 8 individuals (Figure S2C, Supplementary) and, although some genes are expressed only in several individuals and their expression estimates tend to inflate the average R^2 (Figure S2D, Supplementary), their overall impact is negligible due to their small number (similarly to the Smart-Seq2 dataset)."

2) Also in this section, a threshold of 70% average R^2 is used to establish that downsampled data accurately reflect the full data. This threshold seems arbitrary – could the authors provide some justification for this threshold? Figure 1B uses log-transformed transcripts per million (TPM) for mean expression – could the authors justify this choice?

We thank the reviewer for the comment. We would like to point out that 70% is not a specific threshold, but rather an observed value of the average R^2 at coverage 75,000 reads per cell - the value of 70% is chosen arbitrarily. For different levels of coverage, one can observe different levels of the average R^2. We modified the text as follows (Page 4, main text):

"We observe that we can capture most of the expression signal using much lower coverage. For example, 10% of the data ($\approx$75,000 reads per cell) was sufficient to attain $\approx$70% average R^2 across 24,181 genes in alpha cells (Figure 1A)."

Log-transformed TPMs are frequently used in single-cell analysis. We added a reference to the paper "Current best practices in single-cell RNA-seq analysis: a tutorial" by Fabian Theis and modified the text of the manuscript as follows (Page 5, main text):

"log-transformed TPM which is a standard pre-processing for single-cell data[30]"

3) Insufficient technical detail is provided to justify and explain the "effective sample size" concept (N_eff; Pearson R^2 multiplied by sample size), which is used as a key evaluation metric throughout the study. (Two references are provided – refs. 25 and 26 – but these also do not provide much detail.) Since most of the results rely on this metric, it needs to be clearly explained and motivated, including for readers who are not familiar with the concept.

We thank the reviewer for the comment. We do agree that the indicated references do not provide enough details about the effective sample size concept. We included the mathematical proof in the main text of the paper (Page 5):

"Indeed, let y be the ground truth gene expression vector for a given gene G across N individuals (i.e., gene expression obtained at high read coverage) and $\tilde{y}$ be the vector of gene expression estimates obtained at low read coverage of the same gene G across the same N individuals. Let r be the Pearson correlation coefficient between y and $\tilde{y}$. Let g be the genotype at the SNP under testing. For simplicity, all the vectors are standardized with zero mean and unit variance. Let β and $\tilde{\beta}$ be the effect sizes of the SNP in the regression on y and $\tilde{y}$ correspondingly. Regressing y on $\tilde{y}$ we obtain:

$$\tilde{y} = ry + \varepsilon$$

Next, observe that the OLS estimate of $\tilde{\beta}$, $\hat{\tilde{\beta}} = r\hat{\beta}$. Indeed, let ε be noise random variables with mean 0 and variance 1, then:

$$\hat{\tilde{\beta}} = cov(g, \tilde{y}) = cov(g, ry + \varepsilon) = cov(g, ry) + cov(g, \varepsilon) = r\hat{\beta}$$

The standard association test statistics x_{ground} with ground truth estimates is given by the following formula:

$$x_{ground} = Ncor^2(g, y)$$

The association test statistics at low-coverage, $x_{low-coverage}$ is:

$$x_{low-coverage} = Ncor^2(g, \tilde{y}) = N\hat{\tilde{\beta}}^2 = N(r\hat{\beta})^2 = r^2 \cdot Ncor^2(g, y) = \alpha\, x_{ground}$$
”

4) The authors state that a key aim is to provide practical guidelines for designing ct-eQTL studies, and several strong statements are made (e.g. regarding optimal statistical power). However, these results mainly refer to the examples in Figures 2 and 3, which presents specific guidance for these datasets. It is not clear how readers can translate this guidance to new datasets. For example, Figure 2 shows optimal design parameters for the B cell dataset with effective sample size 45. How would readers perform similar design calculations for a new dataset? Would the specific recommendations change for different protocols or different numbers of cells? (These results also rely again on the effective sample size N_eff – how would this be calculated for a new dataset?) Could the authors also provide more details on the definition of optimality, especially in the context of new datasets?

We thank the reviewer for the insightful comment. To facilitate the computations for the readers in case if they apply our guidance to some other datasets, we developed an online web app which takes as parameters budget and sample size and outputs effective sample size and the corresponding number of cells per individual (https://mandricigor.github.io/ct-eqtl-design/).

The 10X dataset used in the manuscript (accession ID: GSE137029) is the largest available dataset in terms of the number of individuals and therefore, it is the best dataset so far which can be used for determining experimental designs with the highest effective sample size across a grid of experiments (we refer to such experimental designs as “optimal”).

With the latest modifications to our simulation pipeline the guidance which we obtained for this dataset can be translated to a new dataset. This guidance is based on the fact that the optimal coverage for ct-eQTL studies is 10,000 reads per cell irrespective of cell type and budget (Figure S14, Supplementary). We obtained this result when answering comment 2 of Reviewer #2 (regarding inaccuracy of demultiplexing at low read coverage). Indeed, when we re-ran the experiments (Figure S10, Supplementary) taking into account not only cell-type misclassification but also demultiplexing inaccuracy at low read coverage, we observed that the optimal read coverage (i.e., read coverage for which the effective sample size was the highest across the grid of experiments) is 10,000 $\pm$ 2,500 reads per cell across all the cell types and budgets (Figures S12 and S13, Supplementary). Indeed, at higher coverages, one wastes budget without significant increases in the effective sample size (shown in Figure 2B), but at lower coverages, demultiplexing inaccuracy and cell-type misclassification prevents one to accurately estimate cell-type-specific gene expression and therefore, lowers the power of ct-eQTL study. Therefore, to translate this guidance to a new dataset, we use 10,000 reads per cell as the optimal level of coverage and compute the corresponding number of cells per individual M (assuming that N is given) using the budget model described in the main text. For each cell type, the effective sample

size at 10,000 reads per cell can be well approximated by a linear function of budget. We added the following text (Page 10, main text):

"Despite the fact that the effective sample size values are different for different cell types across different budgets, the optimal coverage (i.e. coverage, at which we observe the maximum effective sample size across the explored experimental designs) is 10,000 ± 2,500 reads per cell (Figure S13, Supplementary)."

5) Most of the results rely on the use of known reference cell types. However, for many datasets, this information is not available. The authors provide some results on uncertainty due to cell type identification and briefly mention this limitation in the discussion. However, this is a major limitation, which will affect many readers – could the authors expand on this?

We thank the reviewer for the comment. We do agree that label transfer is a reliable technique which allows to accurately annotate cells with cell type labels (even at low coverage, as shown in our paper). In case of missing reference dataset, one has to perform a clustering analysis and identify cell types based on some known marker genes. The impact of low coverage on cell-type identification has been extensively studied in (Pollen et al., 2014) where they showed that cell types can be reliably identified at 10,000 reads per cell. Given the fact that the coverage we recommend for ct-eQTL studies is approximately that value (since for even lower coverage, demultiplexing performs badly, but with a higher coverage the money are being wasted without increasing the effective sample size), this will not have a major impact on the performance of ct-eQTL studies. We modified the text of the paper as follows (Page 13, Discussion):

"In case when a reference dataset is available, one should use the label transfer procedure to accurately annotate cells with cell-type labels. However, even at 10,000 reads per cell, one can reliably identify cell types without any reference dataset[10]."

6) Have the authors considered the use of imputation techniques to further reduce the number of required cells per sample?

We thank the reviewer for the comment. We considered running scImpute tool for single-cell imputation. However, after running scImpute, we observed no increase in the effective sample size for the experiment with the lowest coverage at budget B = $35,000. We added the following text to the manuscript (Page 11, main text):

"Finally, we sought to determine whether imputation tools for scRNA-Seq can increase the effective sample size of ct-eQTL studies. If this would be the case, one could use imputation tools to reduce the number of required cells per sample. We used scImpute[37] to improve the

quality of the dataset with $N = 120$, $M = 2750$, and $r = 2000$ reads per cell at budget $B =$ \$35,000 (the experimental design with the lowest considered coverage) and observed no improvement in the effective sample size. For example, for CD4 T cells, we observed $N_{eff} = 37$ which is equal to the effective sample size for this experimental design without using scImpute (Figure S10, Supplementary). "

7) Code and data to reproduce the results have not been provided. Code and data repositories (e.g. GitHub, Zenodo, etc.) should be provided both for reproducibility purposes and to allow readers to adapt the calculations for new datasets.

We thank the reviewer for the comment. We deposited all the code necessary to reproduce the results of the paper to GitHub: https://github.com/mandricigor/ct-eqtl-design.

Minor comments:

1) When specifying the number of cells per sample, does this refer to the final number of measured cells, or the number of input cells?

We thank the reviewer for the comment. In all our experimental designs, we refer to the number of cells per individual as the final number of measured cells. We modified the text of the paper as follows (Page 7, main text):

"... M is the target number of cells per individual (i.e, final number of measured cells) …"

2) To provide practical guidance for readers, the authors could also consider creating an online calculator, e.g. using R/Shiny.

We thank the reviewer for the idea of a web app. We have created an online calculator similar to the costpercell app from the Satija lab. Our app accepts two parameters: cell type and budget. The output of the calculator is the expected effective sample size and the list of possible experimental designs which can lead to this level of effective sample size. We host our calculator at: https://mandricigor.github.io/ct-eqtl-design/.

3) Figure 1: which cell type is "cell type 1"?

We thank the reviewer for the comment. We modified the text of the manuscript as follows (Page 5, main text):

"average R^2 across 24,181 genes in alpha cells (Figure 1A)"

4) Figure 2: please label panels A and B

We thank the reviewer for the comment. We labeled the panels of Figure 2 with letters A and B.

5) Figure 2B: please provide axis labels

We thank the reviewer for the comment. We provided axis labels - “Budget (thousand dollars)” and “Effective sample size”.

6) Figure 2A: why does the “recommended” design use 33,000 reads per cell, instead of 50,000 as mentioned in the text?

We thank the reviewer for the comment. Based on the minor comment 10 of the Reviewer #2, we updated the word “recommended” to “standard”. Based on the minor comment 7 of the Reviewer #1, we selected sample sizes which are close to “at least 60 individuals”. We also took into account the observation of the reviewer to select an experimental design with the coverage close to 50,000 reads per cell. We updated Figure 2A to take into account the comments of all reviewers. In fact, Figure 2 is now Figure 3.

7) Results, section 2: justification (or a reference) should be provided for the statement “since according to demuxlet, 99% of the sample identities can be correctly recovered at this level of multiplexing”.

We thank the reviewer for the comment. We provided the reference to the demuxlet paper (Kang et al., 2018) (Page 6, main text).

8) Results, section 2: why is the per-reaction capacity limited to 24,000 cells? (e.g. provide reference)

We thank the reviewer for the comment. In the “cell hashing” paper from Satija lab, they “super-loaded” the 10X Genomics instrument such that the expected yield is ~20,000 cells and ~5,000 multiplets. Based on their approach, we chose the maximum value of the expected yield to be 24,000 cells. We included the citation to the “cell hashing” paper when mentioning this threshold in the main text (Page 9, main text):

“We limit the per reaction capacity to 24,000 cells[23] …“

9) Supplementary figures are not referred to in sequence in the text.

We thank the reviewer for the comment. We made sure that the figures were referred to in sequence in the text of the paper.

Reviewers' comments:

Reviewer #1 (Remarks to the Author):

Thank you for your thoughtful responses to my review concerns. I have no further comments.

Reviewer #3 (Remarks to the Author):

The authors have comprehensively addressed the comments raised in the previous version. In particular: (i) the inclusion of an additional 10x Genomics dataset has strengthened the evidence that cell-type-specific gene expression can be accurately inferred at low read coverage, (ii) additional technical detail has been included to explain the "effective sample size" concept, and (iii) providing online code and data along with an online calculator improves reproducibility and makes the results much more accessible and useful for readers.

Reviewer #4 (Remarks to the Author):

Reviewer #2 (new reviewers)

We agree with the original assessment of reviewer #2, that this manuscript will be a valuable resource for the community. The authors generally addressed the comments brought up by reviewer #2 and we think those additional analyses have improved the rigor of the study. However, we have a number of additional comments regarding the metrics used and presentation of the results, the methods used for the variance eQTL study, and the readability of the paper and figures.

Major comments

1. The presentation of results would be improved by showing greater granularity in the results (e.g. showing the distribution of Neff across genes) rather than leaning so heavily on the mean R2 values. This is especially important because, as shown in Figure 1, the standard errors of the average R2 are extremely large for low-coverage datasets, indicating that the distribution of R2 across genes is highly variable – so very different degrees of power will be obtained for different genes. Because the R2 can be calculated for each gene (as in Figure 1), it would be straightforward to ask how many genes achieve Neff greater than some threshold at a given budget/read-depth-per-cell/etc? For example, what budget do I need to have Neff > 70 (GTEx threshold) for at least 5000 genes? How many genes achieve Neff > 70 for the proposed optimal design given a budget of $35,000? Does that budget need to be increased to give truly satisfactory results? When Neff is reported in the results, we suggest that it be phrased as something like "median Neff = 67 (interquartile range 40-95)."

2. The approach used for the variance-eQTL analysis needs much more justification and needs to be explicitly described in the Materials and Methods. Specifically:

a. No methods are provided describing what is done with what data.
b. These results contradict the conclusions of Sarkar et al (PloS Genetics, 2019; https://journals.plos.org/plosgenetics/article?id=10.1371/journal.pgen.1008045) that indicate that huge sample sizes (4000+) are required to have reasonable power to detect variance/dispersion QTLs.
c. Figure 5A (recall for eQTL) and Figure 5B (recall for variance eQTL) look copy pasted. This could be a simple error, or it could be due to the way the variance eQTL analysis was done. The available code (prepare_variance_eqtl_input.py) suggests that the authors computed the variance from log-transformed total-UMI-count-scaled UMI counts. This doesn't account for the mean-variance relationship, so the authors need to provide evidence that the putative variance eQTLs discovered are not simply mean eQTLs that (expected in count data) also affect the observed variance.

3. Throughout the manuscript, the authors compare the down-sampled reads to what they call the "ground truth", which is the full dataset from high-depth Smart-seq2. Even in high-depth Smart-seq2 data, biases exist, meaning this is not ground truth, but rather the best attempt at an accurate per-cell measure of gene expression. This is a reasonable approach for the study, but the nuance should be discussed and the phrase "ground truth" should be replaced with a more specific and accurate term (perhaps "high-coverage").

4. The figures in this manuscript were frequently difficult to interpret or misleading and could be better presented:
a. Mean and standard deviations are shown to summarize results when the distribution (e.g. boxplot/violin plot) of all the data should be presented (Figure 1 and 2).
b. Unclear why Figure 2 is in the main text. While an important point, it doesn't seem to be a focus of the study.
c. A lot of important analysis is "hidden" in the supplemental figures. We recommend the authors:
i. Move results from an example cell type (e.g. dendritic cells) from Figures S4, S5, and S6 up to a main figure.
ii. Move results from an example budget (e.g. $35k) from Figures S8, S9, and S10 up to a main figure.
d. The 3D plots (S4, S5, S6) are difficult to interpret because the differences between the cells are very subtle and it is nearly impossible to see how the shape of the plane differs. Would strongly recommend showing these as heatmap plots with the effective sample size shown using color.
e. The color scales in the heatmaps are not consistent for all panels in a figure and legends are not provided at all, meaning readers have to inspect the (tiny and pixelated) values printed in each cell. This is an issue for Figure 4, S8, S9, and S10. For example, Figure 4 is misleading because it looks like the effect size is independent of cell type frequency.
f. In the heatmaps, zeros are used where NAs are more appropriate (Figures S8, S9, S10): Replace 0's with NA (colored gray) when those zeros are due to budget limitations.
g. In Figure S2A and S2C, the number of individuals in which a gene is expressed is an ordinal, categorical variable – there's no logical reason to connect those points with a line. These panels would be more intuitive if standard boxplots were used.
h. Figures S11 and S12: These figures are hard to interpret because the x-axis is the cell type as a factor and not the cell type composition as a continuous axis. The cell type composition values from Figure S19 should be used for the x-axis, and the cell types should be labeled by color like in S13. For S11 make sample size shape.

Minor comments

1. Recommend adding a link to the webtool to the last sentence of the abstract.
2. Introduction, line 1, para 2: should be "Expression quantitative trait locus [not loci] (eQTL) mapping is a widely-used tool...used...". Further, this sentence implies the utility of eQTL mapping is restricted to understanding genotype-disease associations. This underplays their broad use for understanding genetic regulation of gene expression.
3. Introduction, para 2: could add reference to Cuomo, Seaton, McCarthy, et al, Nat Comms, 2020 to refs 18 and 19, as the latest population-scale single-cell eQTL study.
4. The authors highlight in the discussion that a limitation of this model is that sequencing cost per million reads is a fixed parameter. That is a reasonable limitation for the paper, but the authors could consider allowing users to change this parameter in the web app. This will extend the usefulness of the app as sequencing costs change.
5. Discussion (paragraph 1) claims that the largest available scRNA-Seq dataset with respect to sample size is used, but strictly this is not the case anymore… (Cuomo, Seaton, McCarthy et al Nature Comm 2020) N = 125.
6. Materials and Methods: BS and BS are both used – choose one.

7. Materials and Methods: Bm is not defined beyond f(N, M, x) – please specify the exact functional form(s) used. How was this function derived/generated?
8. Read count simulations: it is stated “the length of vector Rrj is equal to the total number of UMIs in the cell” - my reading is that this should be “total number of genes” from the larger dataset?
9. Cell type classification: it would help the reader to have a brief description of how Seurat’s label transfer method works to provide some intuition for the method without having to look up the paper.
10. Datasets: what genotype data was used for the ct-eQTL analyses? How was it obtained, pre-processed and QC’d before ct-eQTL analysis?
11. It should be specified that costs are provided in US dollars. A reference for the library prep and sequencing costs would also be good.
12. Minor comments regarding the figures:
a. Figure 1C: the y-axis is on a different scale than 1A, B, and D, makes this figure difficult to interpret.
b. Figure 3A: Unclear why the axis labels are in blue. It is also difficult to read the small values inside the lime green boxes. Would recommend using a minimum font size of 6 pts and a less distracting fill color.
c. Figure 3B: The Neff axis label color does not match the line in the figure.
d. In multiple supplemental figures, the average is presented with no standard deviation (Figure S13, S17).
e. Fig S2B and S2D would be easier to read if the raw numbers were shown rather than scientific notation (x10^3).
f. Fig S16 caption: strictly speaking, “reads” are not simulated, rather “read counts” or “UMI counts” - terminology could be tightened up here and elsewhere
g. Typo in caption of Fig S2: second (B) should be (D)
h. It is unclear where the values in Figure S13 come from. If my understanding is correct and the values are the max values from Figure S14, then S14 should come before S13 and the authors should consider making this more explicit (e.g. starring or adding arrows pointing to the values in S14 used in S13).

Christina B. Azodi and Davis J. McCarthy

Response to reviewers for *Mandric et al.*

We thank the reviewers for insightful comments that have allowed us to greatly enhance the quality of our manuscript. You will find that the revised manuscript answers all comments and concerns raised by the reviewer and as such is significantly improved. In particular we have:

- Updated the cell-type-specific variance eQTL pipeline to account for the mean-variance relationship and re-ran the whole set of experiments;
- Re-ran the whole range of simulations at different levels of sequencing cost per 1 million reads and updated the web app;
- Changed all the plots as it was suggested by the reviewer;

For your convenience, we provide a point-by-point response to all comments raised by reviewers in red font below.

Bogdan Pasaniuc (on behalf of all co-authors).

——————-

Reviewer #4

We agree with the original assessment of reviewer #2, that this manuscript will be a valuable resource for the community. The authors generally addressed the comments brought up by reviewer #2 and we think those additional analyses have improved the rigor of the study. However, we have a number of additional comments regarding the metrics used and presentation of the results, the methods used for the variance eQTL study, and the readability of the paper and figures.

Major comments

1. The presentation of results would be improved by showing greater granularity in the results (e.g. showing the distribution of Neff across genes) rather than leaning so heavily on the mean R2 values. This is especially important because, as shown in Figure 1, the standard errors of the average R2 are extremely large for low-coverage datasets, indicating that the distribution of R2 across genes is highly variable – so very different degrees of power will be obtained for different genes. Because the R2 can be calculated for each gene (as in Figure 1), it would be straightforward to ask how many genes achieve Neff greater than some threshold at a given budget/read-depth-per-cell/etc? For example, what budget do I need to have Neff > 70 (GTEx threshold) for at least 5000 genes? How many genes achieve Neff > 70 for the proposed optimal design given a budget of $35,000? Does that budget need to be increased to give truly satisfactory results? When Neff is reported in the results, we suggest that it be phrased as something like “median Neff = 67 (interquartile range 40-95).”

We thank the reviewer for this insightful comment. We showed the distribution of the Pearson R^2 across genes by updating Figure 1 in the main text.

2. The approach used for the variance-eQTL analysis needs much more justification and needs to be explicitly described in the Materials and Methods. Specifically:

a. No methods are provided describing what is done with what data.

We thank the reviewer for this insightful comment. We performed the cell-type-specific variance eQTL analysis the same way we performed the cell-type-specific mean eQTL analysis but with the mean expression as one of the covariates to account for the mean-variance relationship. We updated the text of the paper as follows:

"For the mean and variance ct-eQTL and analysis, we used MatrixEQTL[41] R package. We performed cis-ct-eQTL mapping with cis-distance set to 1 Mbp and p-value threshold set to 5%. As covariates, we used race and disease status. For the variance ct-eQTL analysis, we also used the mean expression as a covariate to account for the mean-variance relationship. The resulting SNP-gene pairs were filtered at an FDR threshold of 5%. The genomic coordinates for each gene were obtained from the GRCh38 genomic annotations downloaded from the Ensemble (release 94)."

b. These results contradict the conclusions of Sarkar et al (PloS Genetics, 2019; https://journals.plos.org/plosgenetics/article?id=10.1371/journal.pgen.1008045) that indicate that huge sample sizes (4000+) are required to have reasonable power to detect variance/dispersion QTLs.

We thank the reviewer for this insightful comment. However, there are a few different reasons for the difference in the results between our manuscript and Sarker et al. First, we would like to point out to the fact that Sarkar et al., 2019 is using a very small single-cell RNA-Seq dataset. Their dataset consists of 53 individuals with 5,447 single cells in total. Therefore, their estimates of the mean and variance of the gene expression for each individual should be extremely noisy. Second, that paper considers a different cell type (iPSC cells). Third, they use a different model for vQTL analysis (and in fact, the paper does not provide any software package to run their code). As opposed to their approach, we considered the variance computed from log-transformed total-UMI-count-scaled UMI counts. We also used ~3 times more variants for the eQTL analyses as compared with Sarkar et al., 2019 (21.4 vs 8.4 million of variants).

c. Figure 5A (recall for eQTL) and Figure 5B (recall for variance eQTL) look copy pasted. This could be a simple error, or it could be due to the way the variance eQTL analysis was done. The available code (prepare_variance_eqtl_input.py) suggests that the authors computed the variance from log-transformed total-UMI-count-scaled UMI counts. This doesn't account for the mean-variance relationship, so the authors need to provide evidence that the putative variance eQTLs discovered are not simply mean eQTLs that (expected in count data) also affect the observed variance.

We thank the reviewer for this insightful comment. We agree with the reviewer that in the previous analysis we did not take care of the mean-variance relationship. We included the mean expression as the covariate in the linear model. We re-ran the whole eQTL analysis pipeline and updated Figure 5.

3. Throughout the manuscript, the authors compare the down-sampled reads to what they call the "ground truth", which is the full dataset from high-depth Smart-seq2. Even in high-depth Smart-seq2 data, biases exist, meaning this is not ground truth, but rather the best attempt at an accurate per-cell measure of gene expression. This is a

reasonable approach for the study, but the nuance should be discussed and the phrase “ground truth” should be replaced with a more specific and accurate term (perhaps “high-coverage”).

We thank the reviewer for this insightful comment. We do agree that the biases in single-cell RNA-Seq technologies prevent us from obtaining the ground truth estimates of the cell-type-specific gene expression. Therefore, we updated the text according to the above comment:

“Due to the biases of single-cell RNA-Seq technologies, the full, high-coverage dataset is not the ground truth, but it can provide the best attempt at an accurate cell-type-specific measure of gene expression. Therefore, throughout the paper, we will refer to it as the “high-coverage” dataset.”

We also replaced the term “ground truth” to “high-coverage” throughout the text of the paper.

4. The figures in this manuscript were frequently difficult to interpret or misleading and could be better presented:
a. Mean and standard deviations are shown to summarize results when the distribution (e.g. boxplot/violin plot) of all the data should be presented (Figure 1 and 2).

We thank the reviewer for this comment. For Figure 1, we updated the plots to boxplots. For Figure 2 (which is now moved to the Supplementary - Figure S8), we do not provide distributions since the plot is based on one 10X reaction.

b. Unclear why Figure 2 is in the main text. While an important point, it doesn’t seem to be a focus of the study.

We thank the reviewer for this comment. Figure 2 is moved to the Supplementary (now Figure S8).

c. A lot of important analysis is “hidden” in the supplemental figures. We recommend the authors:
i. Move results from an example cell type (e.g. dendritic cells) from Figures S4, S5, and S6 up to a main figure.
ii. Move results from an example budget (e.g. $35k) from Figures S8, S9, and S10 up to a main figure.

We thank the reviewer for this comment. We followed the recommendation and moved the results for CD4 T cells from Figures S4-S7 to a main figure (Figure 2). We also moved the results for the budget $35k to a main figure (Figure 3).

d. The 3D plots (S4, S5, S6) are difficult to interpret because the differences between the cells are very subtle and it is nearly impossible to see how the shape of the plane differs. Would strongly recommend showing these as heatmap plots with the effective sample size shown using color.

We thank the reviewer for this comment. We replaced the 3D plots with the heatmaps where the effective sample size is shown using color. The corresponding figures are: Figures S4 - S7.

e. The color scales in the heatmaps are not consistent for all panels in a figure and legends are not provided at all, meaning readers have to inspect the (tiny and pixelated) values printed in each cell. This is an issue for Figure 4, S8,

S9, and S10. For example, Figure 4 is misleading because it looks like the effect size is independent of cell type frequency.

We thank the reviewer for this comment. We have updated the heatmaps so that they are consistent for all panels in the figures.

f. In the heatmaps, zeros are used where NAs are more appropriate (Figures S8, S9, S10): Replace 0's with NA (colored gray) when those zeros are due to budget limitations.

We thank the reviewer for this comment. We replaced 0's with NA and changed the colors of the corresponding cells to gray. The corresponding heatmaps are now Figures S9, S10, S11.

g. In Figure S2A and S2C, the number of individuals in which a gene is expressed is an ordinal, categorical variable – there's no logical reason to connect those points with a line. These panels would be more intuitive if standard boxplots were used.

We thank the reviewer for this comment. We have updated the figure S2 to reflect the reviewer's suggestions.

h. Figures S11 and S12: These figures are hard to interpret because the x-axis is the cell type as a factor and not the cell type composition as a continuous axis. The cell type composition values from Figure S19 should be used for the x-axis, and the cell types should be labeled by color like in S13. For S11 make sample size shape.

We thank the reviewer for this insightful comment. For Figure S11, we made the x-axis to be sample size and for Figure S12, we made the x-axis to be cell type abundance. In both figures, cell types are labeled by color the same way as in Figure S13.

Minor comments

1. Recommend adding a link to the webtool to the last sentence of the abstract.

We thank the reviewer for this insightful comment. We modified the text of the abstract as follows:

Finally, we provide a practical approach on selecting cost-effective designs for maximizing cell-type-specific eQTL power which is available at https://mandricigor.github.io/ct-eqtl-design/ in the form of a web tool.

2. Introduction, line 1, para 2: should be "Expression quantitative trait locus [not loci] (eQTL) mapping is a widely-used tool...used...". Further, this sentence implies the utility of eQTL mapping is restricted to understanding genotype-disease associations. This underplays their broad use for understanding genetic regulation of gene expression.

We thank the reviewer for this insightful comment. We modified the text as follows:

"Expression quantitative trait locus (eQTL) mapping is a widely-used tool in functional genomics used to identify mechanisms underlying the genotype-to-disease connection[14,15] and genetic regulation of gene expression[16,17]."

In fact, we added the references to two papers - (Fagny et al., 2017) and (Keele et al., 2020).

3. Introduction, para 2: could add reference to Cuomo, Seaton, McCarthy, et al, Nat Comms, 2020 to refs 18 and 19, as the latest population-scale single-cell eQTL study.

We thank the reviewer for this comment. We added the reference to Cuomo, Seaton, McCarthy, et al.

4. The authors highlight in the discussion that a limitation of this model is that sequencing cost per million reads is a fixed parameter. That is a reasonable limitation for the paper, but the authors could consider allowing users to change this parameter in the web app. This will extend the usefulness of the app as sequencing costs change.

We thank the reviewer for this comment. We updated our web app by adding the parameter representing the sequencing cost per 1 million reads.

5. Discussion (paragraph 1) claims that the largest available scRNA-Seq dataset with respect to sample size is used, but strictly this is not the case anymore… (Cuomo, Seaton, McCarthy et al Nature Comm 2020) N = 125.

We thank the reviewer for this comment. In fact, we used the dataset that consists of 142 individuals. Out of these, we selected 120 individuals that had at least 3000 cells. This gave us enough freedom when sampling cells for each considered experimental design.

6. Materials and Methods: BS and BS are both used – choose one.

We thank the reviewer for the comment. We used B^s only once - as the cost of sequencing.

7. Materials and Methods: Bm is not defined beyond f(N, M, x) – please specify the exact functional form(s) used. How was this function derived/generated?

We thank the reviewer for this comment. We used the implementation of this function based on the Satija lab single-cell cost calculator. We updated the text of the paper as follows:

“The function is implemented based on the code of the Satija lab single-cell cost calculator (https://satijalab.org/costpercell).”

8. Read count simulations: it is stated “the length of vector Rrj is equal to the total number of UMIs in the cell” - my reading is that this should be “total number of genes” from the larger dataset?

We thank the reviewer for this comment. The length of the vector R_j^r is equal to the number of UMIs and not the number of genes. We are simulating read counts for each UMI: if there is at least one read for a UMI then this UMI is detected, otherwise not.

9. Cell type classification: it would help the reader to have a brief description of how Seurat's label transfer method works to provide some intuition for the method without having to look up the paper.

We thank the reviewer for this comment. To improve the readability of the paper and to make the reading smooth (i.e, without the need to consult the corresponding paper with the label transfer procedure), we modified the text of the paper as follows:

"Label transfer is a two-stage approach based on the paradigm of mutual nearest neighbor (MNN) matching between a query and a reference dataset. In the first stage, a set of anchor query-reference pairs is identified. Anchors represent cell pairs with highly similar state cells across the datasets. The cell types of the cells in each pair are supposed to be the same. In the second stage, the prediction of the cell-type label of each non-anchor cell in the query dataset is performed based on an appropriate weighting scheme between each anchor and each non-anchor cell."

10. Datasets: what genotype data was used for the ct-eQTL analyses? How was it obtained, pre-processed and QC'd before ct-eQTL analysis?

We thank the reviewer for this insightful comment. We updated the text of the paper with the following details:

"The individuals were genotyped on the Affymetrix World LAT Array. The raw intensity .CEL files were processed according to the APT best practices using the Analysis Power Tools command line utility (APT). Poor quality probe sets were removed using APT's SNPpolisher functionality. Resulting genotypes were used for imputation with the Haplotype Reference Consortium version 1.1 and a MAF < 0.01. The genotypes were imputed using Minimac3. The total of 21,412,068 variants were used in the analysis."

11. It should be specified that costs are provided in US dollars. A reference for the library prep and sequencing costs would also be good.

We thank the reviewer for this comment. We indicated that the costs are provided in the US dollars. We modified the text as follows:

To evaluate this relationship in realistic settings, which includes the number of cells per individual and sample preparation cost, we model the budget (in US dollars) as:

$$B = B^m + \frac{N \cdot L}{x} + (10^{-6} \cdot p) \cdot N \cdot M \cdot r,$$

where N is the sample size, M is the target number of cells per individual (i.e, final number of measured cells) , r is the read coverage, and x is the degree of sample multiplexing (number of individuals per reaction). p is the average cost of Illumina sequencing per 1 million reads (in US dollars), L is the library preparation cost per reaction (in US dollars), and B^m is the budget (in US dollars) wasted on sequencing of identifiable multiplets.

12. Minor comments regarding the figures:
a. Figure 1C: the y-axis is on a different scale than 1A, B, and D, makes this figure difficult to interpret.

We thank the reviewer for this comment. We fixed the issue.

b. Figure 3A: Unclear why the axis labels are in blue. It is also difficult to read the small values inside the lime green boxes. Would recommend using a minimum font size of 6 pts and a less distracting fill color.

We thank the reviewer for this comment. We updated the axis labels to black, changed the font size to 7 pts, and chose a less distracting fill color. Note that Figure 3 is now Figure 4.

c. Figure 3B: The Neff axis label color does not match the line in the figure.

We thank the reviewer for this comment. We fixed the color.

d. In multiple supplemental figures, the average is presented with no standard deviation (Figure S13, S17).

We thank the reviewer for this comment. The numbers in Figure S13 are the averages of 3 values and therefore, we do not provide standard deviations. We updated Figure S17 by adding standard deviations to the plot.

e. Fig S2B and S2D would be easier to read if the raw numbers were shown rather than scientific notation (x10^3).

We thank the reviewer for this comment. We updated Figures S2B and S2D.

f. Fig S16 caption: strictly speaking, “reads” are not simulated, rather “read counts” or “UMI counts” - terminology could be tightened up here and elsewhere.

We thank the reviewer for this comment. We fixed the caption of Figure S16.

g. Typo in caption of Fig S2: second (B) should be (D)

We thank the reviewer for this comment. We fixed the typo.

h. It is unclear where the values in Figure S13 come from. If my understanding is correct and the values are the max values from Figure S14, then S14 should come before S13 and the authors should consider making this more explicit (e.g. starring or adding arrows pointing to the values in S14 used in S13).

We thank the reviewer for this comment. It is right that the values in Figure S13 come from Figure S14. In fact, both the values in Figures S13 and S14 come from Figure 10. Moreover, Figure S13 is referenced earlier in the main text. Therefore, we left the figures in their original order.

REVIEWERS' COMMENTS:

Reviewer #4 (Remarks to the Author):

The authors have thoughtfully responded to all of our suggestions and comments and have updated analyses and figures where appropriate.

Our only additional comment is that we would encourage the authors to include the explanation they provided us in response to major comment 2b in the discussion of the manuscript. Given the difficulties with variant-QTL mapping demonstrated by Sarkar et al (2019), we believe a brief discussion of why v-QTL mapping was successful here would be useful to readers.

We congratulate the authors on a useful and interesting paper.

Christina B. Azodi and Davis J. McCarthy

Response to reviewers for *Mandric et al.*

We thank the reviewers for insightful comments that have allowed us to greatly enhance the quality of our manuscript. You will find that the revised manuscript answers all comments and concerns raised by the reviewer. For your convenience, we provide a point-by-point response to all comments raised by reviewers in red font below.

Bogdan Pasaniuc (on behalf of all co-authors).

——————-

Reviewer #4

1. The authors have thoughtfully responded to all of our suggestions and comments and have updated analyses and figures where appropriate.
Our only additional comment is that we would encourage the authors to include the explanation they provided us in response to major comment 2b in the discussion of the manuscript. Given the difficulties with variant-QTL mapping demonstrated by Sarkar et al (2019), we believe a brief discussion of why v-QTL mapping was successful here would be useful to readers.
We congratulate the authors on a useful and interesting paper.
Christina B. Azodi and Davis J. McCarthy.

We thank the reviewers for a comprehensive review. We updated the text of the manuscript according to their suggestions (Discussion section):

Fourth, the numbers of variance QTLs that we found in this study do not agree with the conclusions of a recent study that large sample sizes (over 4000) are required to have reasonable power to detect variance QTLs[41]. There are three reasons for the difference between our results and (41). First, (41) used a very small single-cell RNA-Seq dataset. Their dataset consists of 53 individuals with 5,447 single cells in total. Therefore, their estimates of the mean and variance of the gene expression for each individual should be extremely noisy. Second, that paper considers a different cell type (iPSC cells). Third, a different model for variance QTL analysis was used in (41). As opposed to their approach, we considered the variance computed from log-transformed total-UMI-count-scaled UMI counts. We also used ~3 times more variants for the eQTL analyses as compared with (41) (21.4 vs 8.4 million of variants).
